# Supplementary material for: Structural insights into lipid membrane binding by human ferlins
Source: EMBO J. 2025 May 28;44(14):3926–58. doi: 10.1038/s44318-025-00463-8 (PMC12264198; doi:10.1038/s44318-025-00463-8)
Supplement: Supplementary file 1 — Appendix [file 44318_2025_463_MOESM1_ESM.pdf]

Appendix

for **“Structural insights into lipid membrane binding by human ferlins”**

Table of Contents

Appendix Figure S1 2

Appendix Figure S2 4

Appendix Figure S3 6

Appendix Figure S4 8

Appendix Figure S5 10

Appendix Figure S6 12

Appendix Figure S7 14

Appendix Figure S8 16

Appendix Figure S9 18

Appendix Figure S10 20

Appendix Figure S11 22

Appendix Figure S12 24

Appendix Figure S13 26

Appendix Figure S14 28

Appendix Figure S15 30

Appendix Table S1 32

Appendix Table S2 33

Appendix Table S3 34

Appendix Table S4 35

Appendix Table S5 36

Appendix Table S6 37

Appendix Table S7 38

References 38

Appendix Figure S1

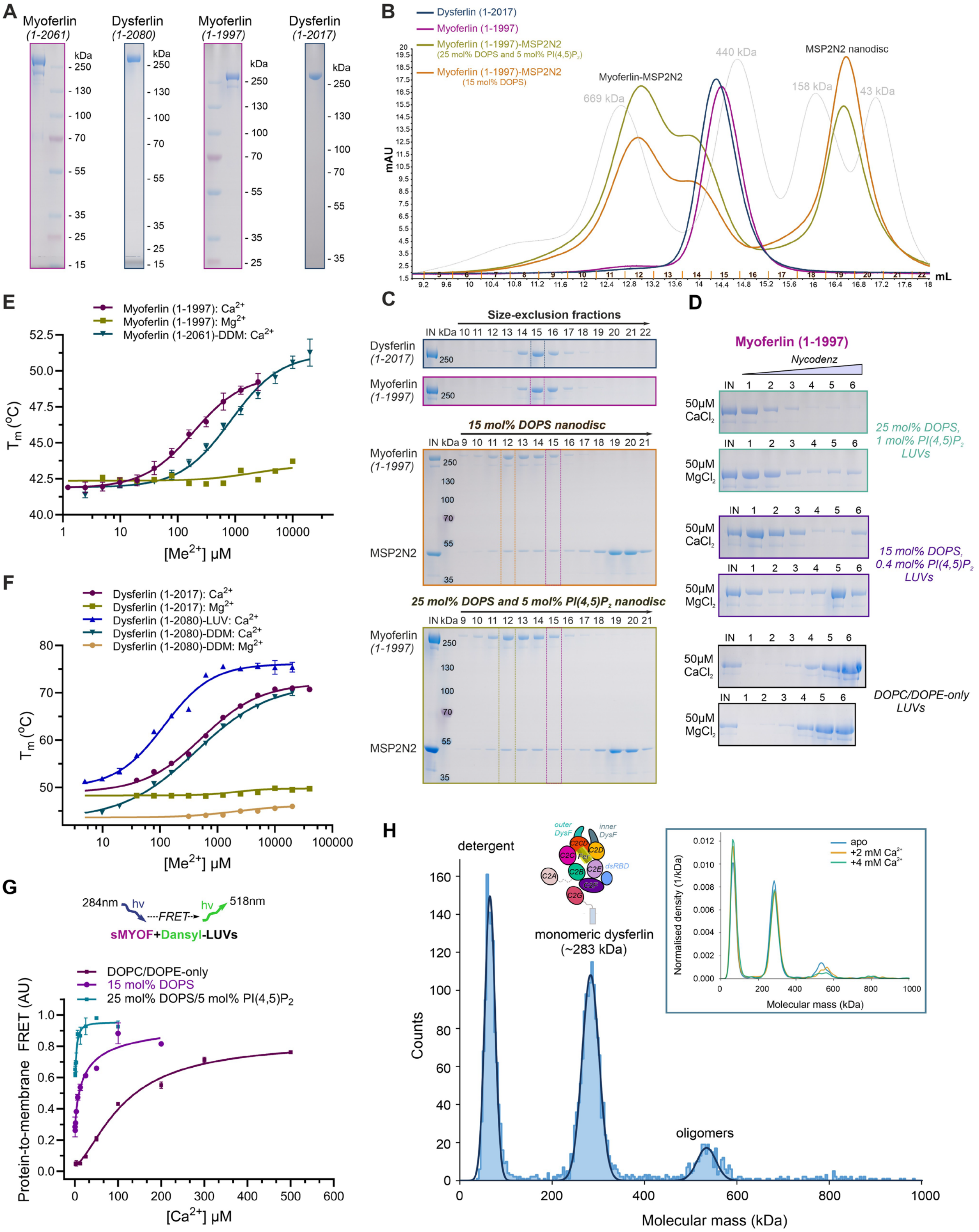

Appendix Figure S1. Biochemical characterization of human myoferlin and dysferlin.

A SDS-PAGE analysis of purified myoferlin and dysferlin constructs, used for structural and functional studies.

B Size-exclusion chromatography (SEC) elution profile of the soluble myoferlin (residues 1-1997, sMYOF), soluble dysferlin (residues 1-2017, sDYSF), and the reconstituted myoferlin (1-1997)-MSP2N2 complexes. MSP2N2-based nanodiscs, comprising 15 mol% DOPS or 25 mol% DOPS and 5 mol% PI(4,5)P_2 anionic phospholipids, were purified through SEC prior to the assembly of their complexes with sMYOF. Gel filtration standards (grey) were applied to the Superose 6 column in the same buffer and their molecular masses are indicated.

C SDS-PAGE analysis of the size-exclusion chromatography peak fractions from B. To enable compact visualization of the myoferlin (1-1997) and dysferlin (1-2017) fractions, the top two gels were cropped (see also S1A and Source Data).

D Ca^{2+}-sensitive liposome binding activity of soluble myoferlin (1-1997) using a coflotation assay. Myoferlin-liposome (large unilamellar vesicles, LUVs) complexes were assembled *in vitro* in the presence of 50 µM Ca^{2+} or Mg^{2+} and subjected to ultracentrifugation on a Nycodenz step gradient (0%/30%/40%). The Nycodenz gradients were harvested from the top. The anionic phospholipid compositions of the tested LUVs are indicated. LUVs without anionic phospholipids are referred to as "DOPC/DOPE-only LUVs".

E Ca^{2+}-binding activity of membrane-anchored, full-length myoferlin (residues 1-2061, in detergent micelles, DDM) and soluble myoferlin (residues 1-1997) analysed using a nanoDSF-based assay. Error bars represent the standard error of the mean (s.e.m.; soluble myoferlin (1-1997): n=4, full-length myoferlin (1-2061)-DDM: n=7). $[Ca^{2+}]_{1/2}$ was estimated by nonlinear regression curve fitting and Mg^{2+} titration was used as a control.

F Ca^{2+}-binding activity of full-length dysferlin (residues 1-2080, in detergent micelles, DDM), soluble dysferlin (residues 1-2017), and dysferlin proteoliposomes (dysferlin (1-2080)-LUVs) analysed using a nanoDSF-based assay. Dysferlin proteoliposomes were prepared by dialysis of the full-length sample in the presence of extruded LUVs (composed of 70 mol% DOPC, 20 mol% DOPE and 10 mol% cholesterol). The error bars represent the s.e.m. (dysferlin (1-2017): n=4, dysferlin (1-2080)-DDM: n=6, dysferlin (1-2080)-LUV: n=6).

G Ca^{2+}-sensitive liposome binding activity of soluble myoferlin (residues 1-1997, sMYOF) using a fluorescence-based lipid-binding assay. Dansyl-labelled LUVs, prepared with DOPS (15 mol% DOPS), DOPS and PI(4,5)P_2 (25 mol% DOPS and 5 mol% PI(4,5)P_2) or without anionic phospholipids (DOPC/DOPE-only), were used at a 50 µM concentration. Error bars represent the s.e.m. (n=3).

H Mass-photometry characterization of full-length dysferlin (residues 1-2080) reconstituted in detergent micelles (LMNG), showing a major population of monomeric dysferlin particles (molar mass of ~283 kDa). The inset depicts the mass distributions of dysferlin's major species at two Ca^{2+} concentrations (2 mM $CaCl_2$ and 4 mM $CaCl_2$, orange and green curves, respectively), compared to the Ca^{2+}-free state (blue). In all cases, the mass photometry measurements were conducted at a ~50 nM dysferlin concentration in a sample buffer devoid of detergent. A domain composition schematic is shown above the mass photometry profile. The mass photometry measurements were repeated four times (n=4).

Appendix Figure S2

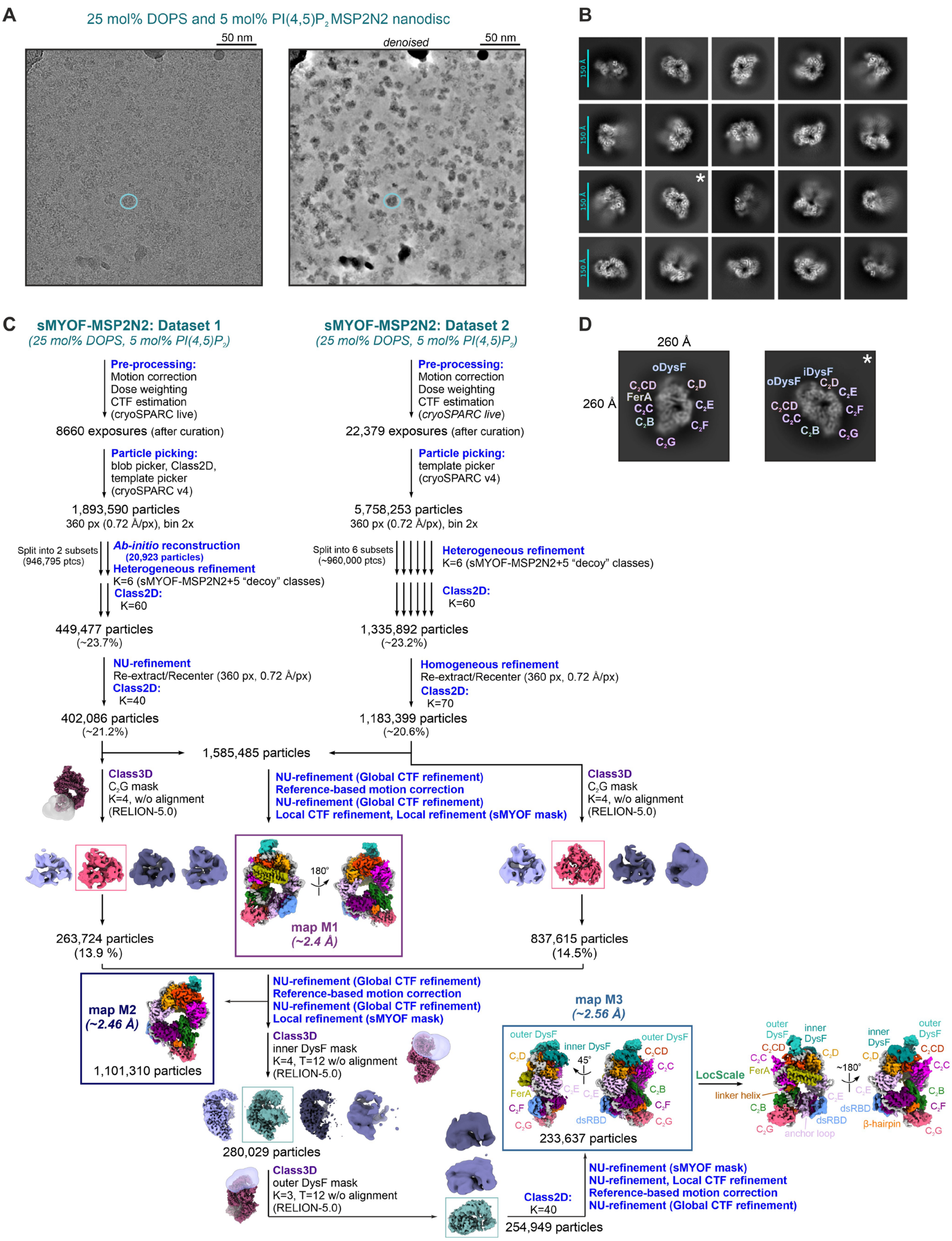

Appendix Figure S2. Computational image analysis of the vitrified myoferlin (1-1997)-nanodisc complex (25 mol% DOPS and 5 mol% PI(4,5)P_2 nanodisc).

A Cryo-EM micrograph of the vitrified myoferlin (1-1997)-nanodisc complex (25 mol% DOPS and 5 mol% PI(4,5)P_2 MSP2N2 nanodisc). A characteristic, monomeric particle is circled in cyan, in both the raw (left) and denoised (right) exposure.

B Reference-free 2D class averages of the myoferlin (1-1997)-nanodisc complex, comprising 25 mol% DOPS and 5 mol% PI(4,5)P_2. High-resolution 2D class averages of the sample were generated with the cryosparc-tools package (https://tools.cryosparc.com/examples/hi-res-2d-classes.html). The 2D average marked with an asterisk is shown as a close-up view in D.

C Cryo-EM data processing routine of the lipid-bound myoferlin, assembled on a 25 mol% DOPS and 5 mol% PI(4,5)P_2 MSP2N2 nanodisc (see also Appendix Fig S1B-C). The final maps are boxed and colour-coded after the modelled domains (as in Fig 1).

D Close-up views of two characteristic 2D class averages of the myoferlin (1-1997)-MSP2N2 complex (25 mol% DOPS and 5 mol% PI(4,5)P_2 nanodisc). The visible myoferlin domains are indicated and colour-coded.

Appendix Figure S3

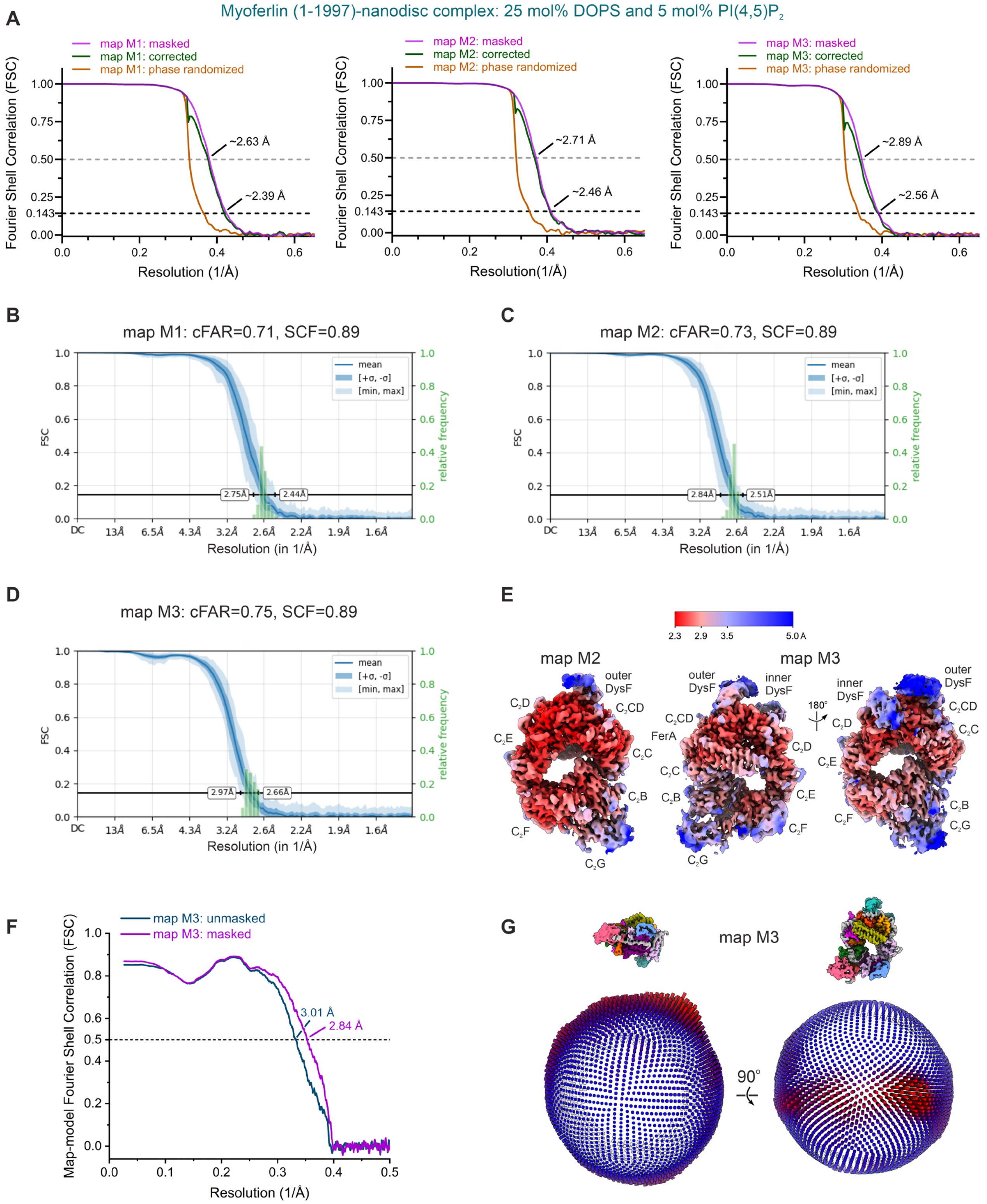

Appendix Figure S3. Resolution and quality of the lipid-bound myoferlin (1-1997) cryo-EM maps (25 mol% DOPS and 5 mol% PI(4,5)P_2 nanodisc).

A Fourier shell correlation (FSC) between the final myoferlin (1-1997)-nanodisc cryo-EM half-maps (25 mol% DOPS and 5 mol% PI(4,5)P_2) MSP2N2 nanodisc). The gold-standard FSC criterion (FSC=0.143) indicates global resolution estimates of ~2.39 Å (consensus map), ~2.46 Å (after masked C_2G classification), and ~2.56 Å (after masked DysF classification) for the M1, M2, and M3 maps, respectively.

B-D Directional resolution and Fourier sampling of the myoferlin (1-1997)-nanodisc cryo-EM maps. The conical FSC, the conical FSC area ratio (cFAR), and the sampling compensation factor (SCF) were calculated and plotted with cryoSPARC v.4.5.

E Local resolution of the myoferlin (1-1997)-nanodisc cryo-EM maps (25 mol% DOPS and 5 mol% PI(4,5)P_2 MSP2N2 nanodisc). Red colour indicates higher resolution.

F Map versus model FSC plot for the myoferlin (1-1997)-nanodisc complex (map M3, 25 mol% DOPS and 5 mol% PI(4,5)P_2 MSP2N2 nanodisc).

G 3D angular distribution of the myoferlin particles contributing to the overall (M3) map (25 mol% DOPS and 5 mol% PI(4,5)P_2) MSP2N2 nanodisc). The similarly oriented and colour-coded maps of the complex are shown above the angular distribution plot. Red colour and relative cylinder height indicate a higher number of particle images.

Appendix Figure S4

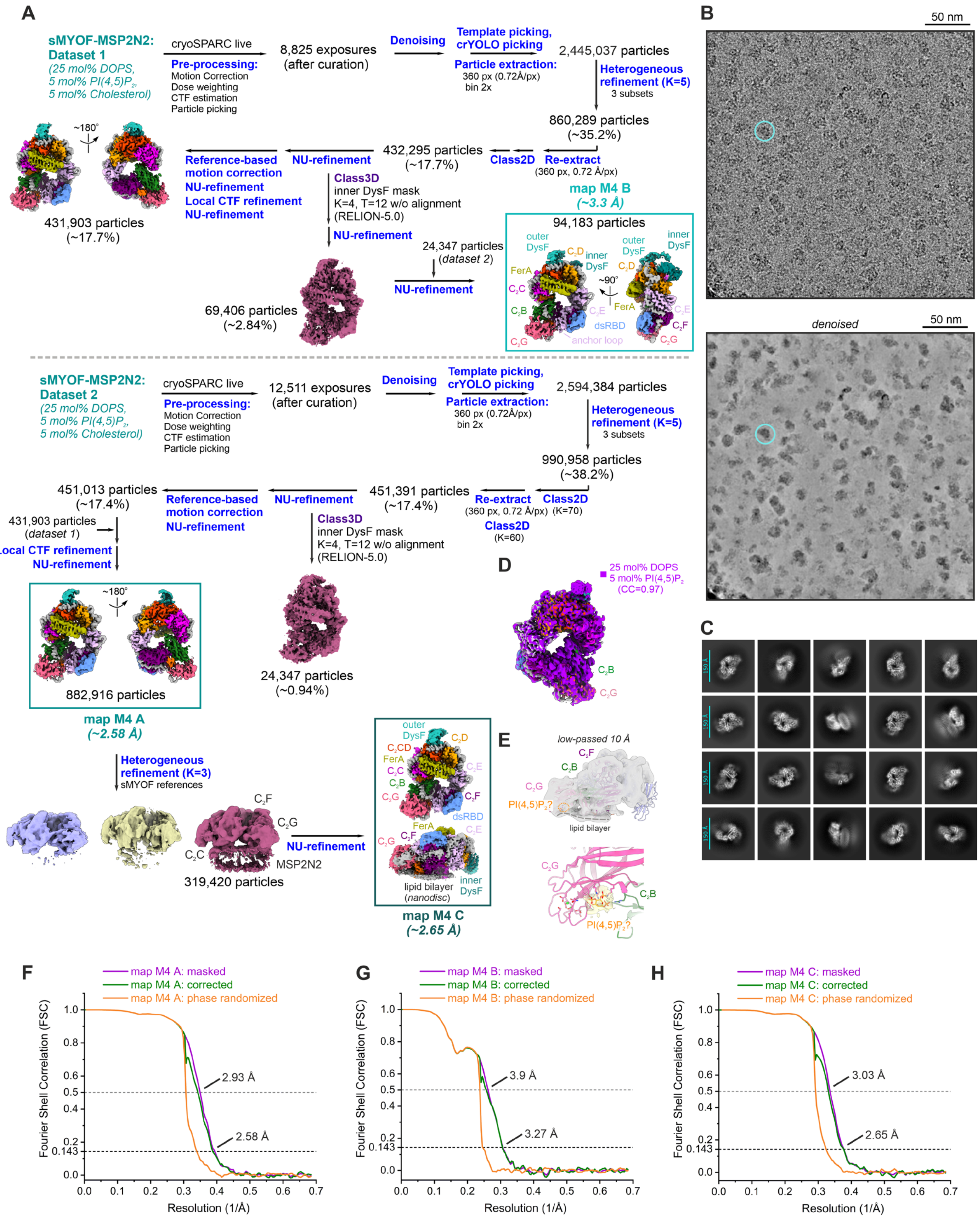

Appendix Figure S4. Cryo-EM image analysis of the vitrified myoferlin (1-1997)-nanodisc complex (25 mol% DOPS, 5 mol% PI(4,5)P_2 and 5 mol% cholesterol MSP2N2 nanodisc).

A Cryo-EM processing schematic for the soluble myoferlin (1-1997), bound to a 25 mol% DOPS, 5 mol% PI(4,5)P_2 and 5 mol% cholesterol MSP2N2 nanodisc. The resolution estimates (FSC=0.143 criterion) of the final maps are indicated and the final maps are coloured after the modelled domains.

B Cryo-EM micrograph (raw and denoised) of the imaged myoferlin (1-1997)-nanodisc complex (containing 25 mol% DOPS, 5 mol% PI(4,5)P_2 and 5 mol% cholesterol). A single vitrified particle is circled in cyan.

C Reference-free 2D class averages of the myoferlin (1-1997)-nanodisc complex (25 mol% DOPS, 5 mol% PI(4,5)P_2 and 5 mol% cholesterol nanodisc). Images of 2D class averages were generated with the cryosparc-tools package (https://tools.cryosparc.com/examples/hi-res-2d-classes.html).

D Superposition between the consensus cryo-EM maps of nanodisc-bound myoferlin (1-1997), obtained in the absence (purple, 25 mol% DOPS and 5 mol% PI(4,5)P_2) or presence of cholesterol (colour-coded, 25 mol% DOPS, 5 mol% PI(4,5)P_2 and 5 mol% cholesterol).

E Low-passed cryo-EM map of the lipid-bound myoferlin (25 mol% DOPS, 5 mol% PI(4,5)P_2 and 5 mol% cholesterol nanodisc, map M4 C) showing ordered lipid headgroups, including a tentative PI(4,5)P_2, located at the interface between C_2F, C_2G and C_2B.

F-H Fourier shell correlation (FSC) plots for the myoferlin (1-1997)-nanodisc cryo-EM maps (25 mol% DOPS, 5 mol% PI(4,5)P_2 and 5 mol% cholesterol datasets).

Appendix Figure S5

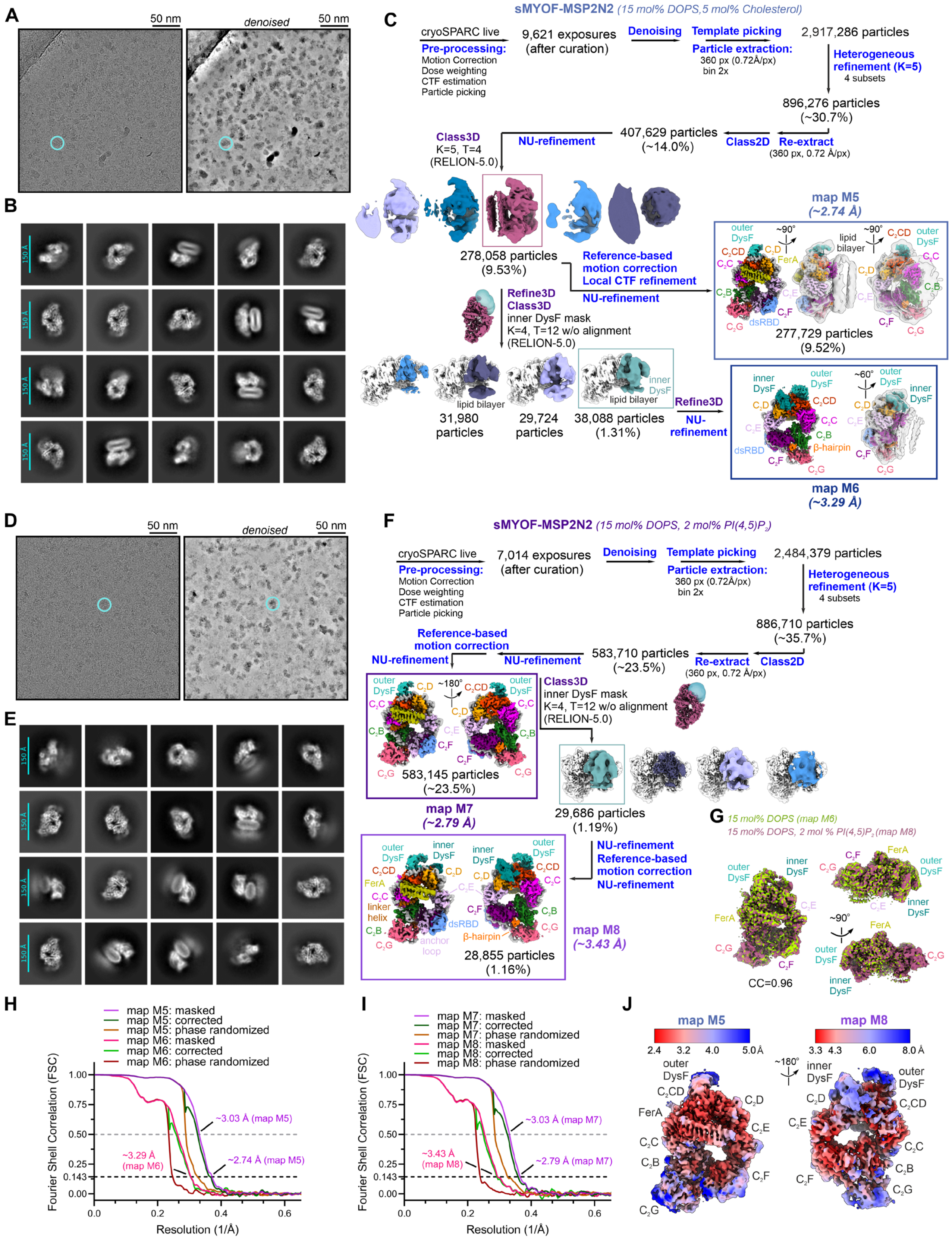

Appendix Figure S5. Cryo-EM analysis of the myoferlin (1-1997)-lipid complexes assembled on 15 mol% DOPS and 5 mol% Cholesterol or 15 mol% and 2 mol% PI(4,5)P_2 MSP2N2 nanodiscs.

A-B Cryo-EM (raw and denoised) micrograph and 2D class averages of the myoferlin (1-1997)-nanodisc complex (15 mol% DOPS and 5 mol% cholesterol MSP2N2 nanodisc). The 2D class averages shown in B were generated with the cryosparc-tools package (https://tools.cryosparc.com/examples/hi-res-2d-classes.html).

C Cryo-EM data processing schematic for vitrified myoferlin (1-1997) bound to a 15 mol% DOPS and 5 mol% cholesterol nanodisc. The final maps are coloured according to the modelled domains and the resolution estimates (FSC=0.143 criterion) are indicated.

D-F Cryo-EM image analysis of the soluble myoferlin (1-1997)-lipid complex, assembled on a 15 mol% DOPS and 2 mol% PI(4,5)P_2 MSP2N2 nanodisc. The 2D class averages shown in E were generated with the cryosparc-tools package (https://tools.cryosparc.com/examples/hi-res-2d-classes.html).

G Superposition between the M6 (15 mol% DOPS and 5 mol% Cholesterol) and M8 (15 mol% DOPS and 2 mol% PI(4,5)P_2) nanodisc-bound myoferlin (1-1997) maps. The two cryo-EM maps are nearly identical (CC=0.96).

H-I Fourier shell correlation (FSC) plots for the lipid-bound myoferlin (1-1997) maps (15 mol% DOPS and 2 mol% PI(4,5)P_2 or 15 mol% DOPS and 5 mol% cholesterol nanodisc complexes).

J Local resolution of the myoferlin (1-1997)-nanodisc cryo-EM maps, depicted in C and F. Red colour indicates a higher resolution.

Appendix Figure S6

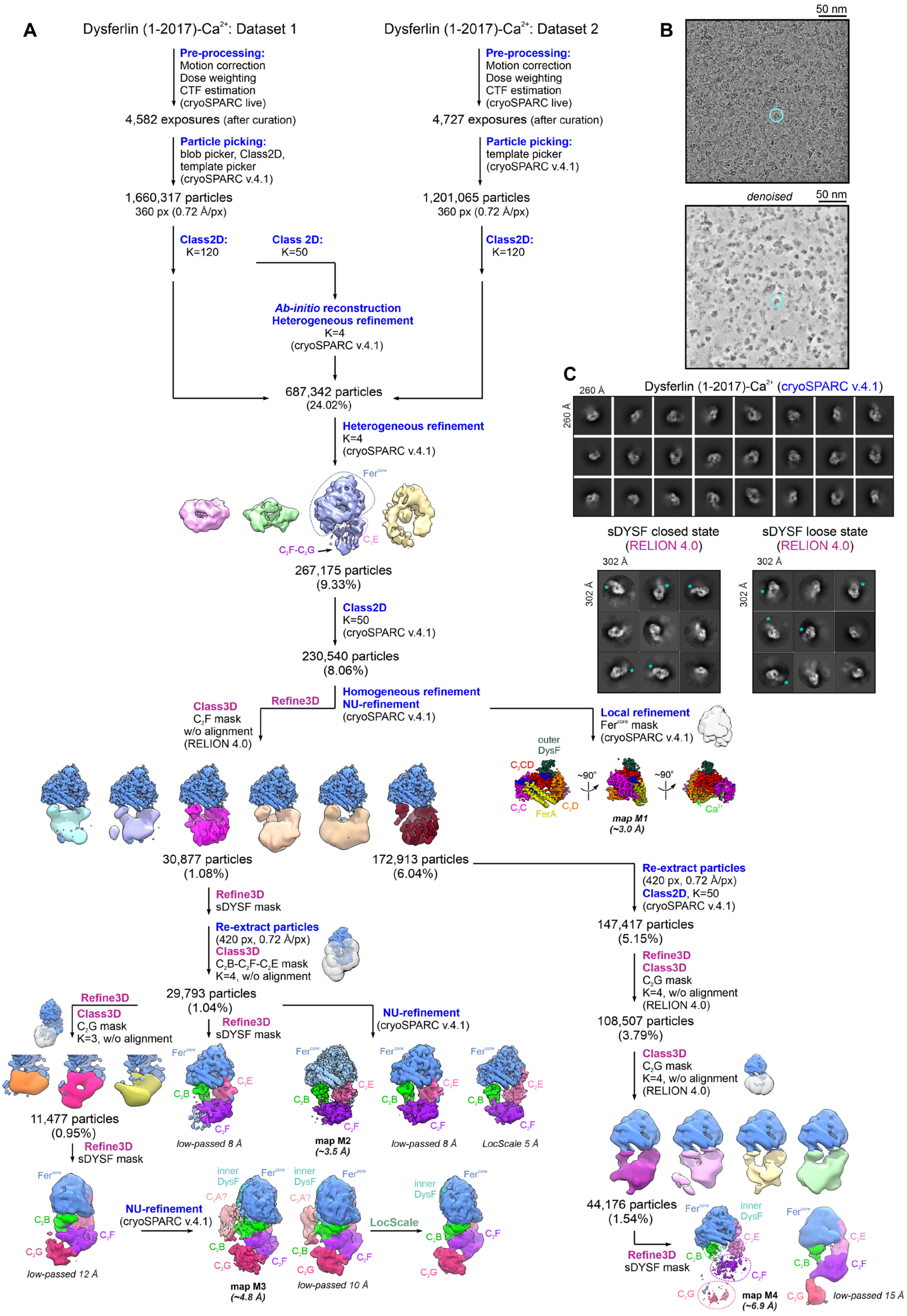

Appendix Figure S6. Computational image analysis of the lipid-free soluble dysferlin (1-2017) cryo-EM datasets.

A Cryo-EM data processing schematic for the Ca^{2+}-bound soluble dysferlin (sDYSF, residues 1-2017). The resolution of the final maps (M1-M4) was estimated according to the gold-standard Fourier Shell Correlation (FSC) cutoff of 0.143. The key structural domains of dysferlin (1-2017) are coloured and indicated in the final maps.

B Typical cryo-EM micrograph (raw and denoised) of the vitrified lipid-free soluble dysferlin (1-2017). A dysferlin particle is circled in cyan.

C Reference-free 2D class averages of lipid-free dysferlin (1-2017). The 2D class averages calculated from the "closed" and "loose" state particle images are shown in the bottom panel. The C_2F domain is marked with an asterisk. The C-terminal domain is more ordered in the "closed" state 2D classes (left panel) compared to the "loose" dysferlin state (right panel).

Appendix Figure S7

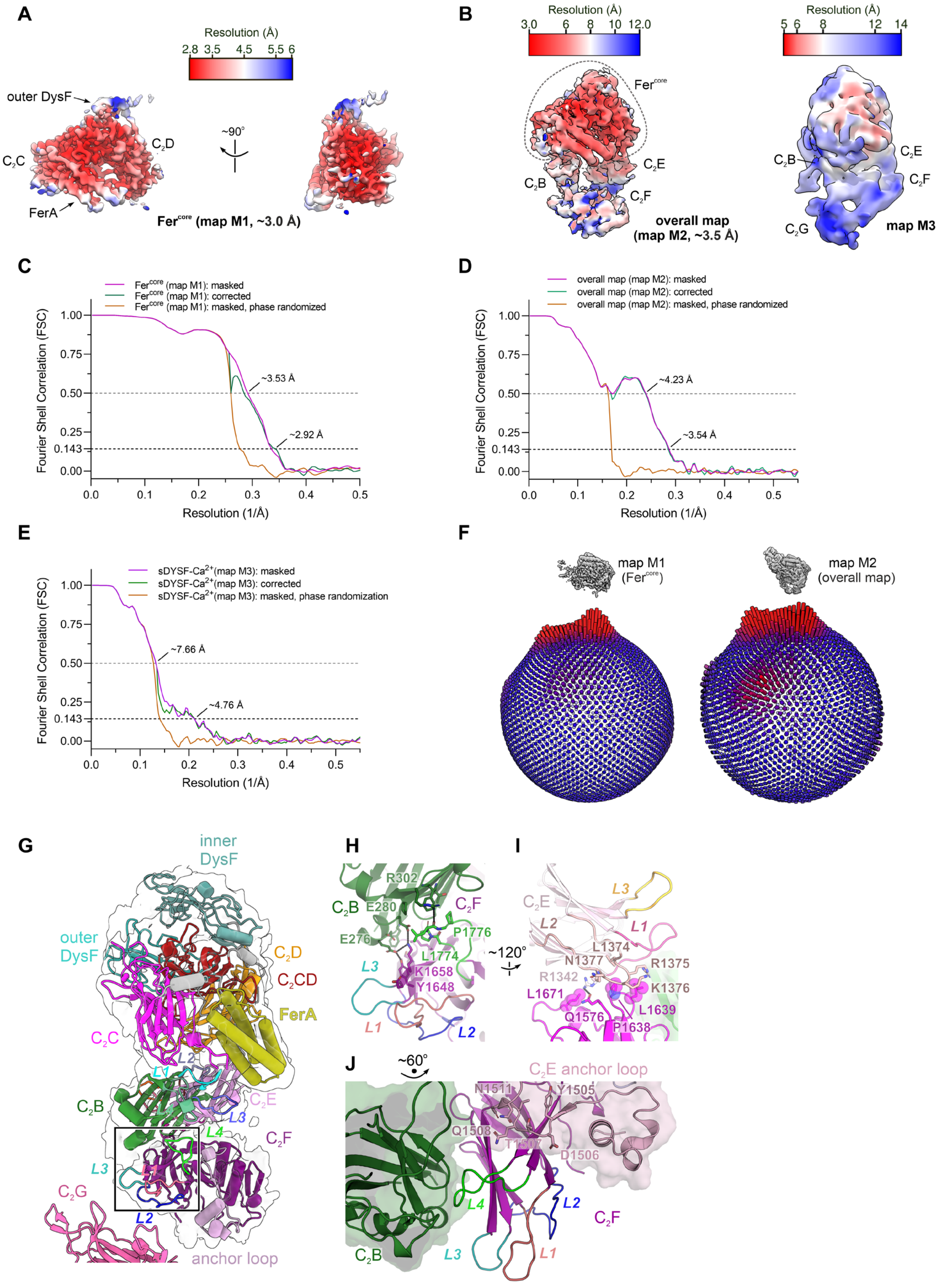

Appendix Figure S7. Resolution and quality of the lipid-free dysferlin (1-2017) cryo-EM maps.

A Local resolution of the lipid-free Fer^{core} map (map M1) of dysferlin (1-2017). Red colour indicates higher resolution.

B Overall maps of Ca^{2+}-bound soluble dysferlin (map M2 and M3), coloured by local resolution. The Fer^{core} (C_2C-C_2CD-FerA-C_2D) is resolved at a higher resolution compared to the more dynamic C_2B-C_2F-C_2E region.

C-E. Fourier shell correlation (FSC) between the final lipid-free dysferlin (1-2017) half-maps. The gold-standard FSC criterion (FSC=0.143) indicates global resolution estimates of ~2.9 Å, ~3.5 Å, and ~4.8 Å for the M1, M2, and M3 maps, respectively.

F 3D angular distribution of the dysferlin particles contributing to the Fer^{core} (M1) and overall (M2) maps. Red colour and increased cylinder height indicate a higher number of particle images.

G Tertiary interfaces between C_2F, C_2B, and C_2E observed in the dysferlin (1-2017) cryo-EM model. In the lipid-free state, the C_2F domain is positioned at the base of the dysferlin ring through multiple interactions with the N-terminal C_2B and upstream C_2E. The final model of dysferlin (1-2017) is shown as cartoons and fitted inside the overall cryo-EM map (transparent surface). The L1-L4 loops of C_2F are coloured pink, blue, teal, and green, respectively.

H Selected polar interactions between C_2F's L3-L4 loops and C_2B. The polar contacts between interacting residues are shown as dashed lines.

I Polar and hydrophobic interactions between the bottom loops of C_2F and the L2 loop of C_2E. Residues involved in hydrophobic interactions are shown as transparent spheres.

J Contact interfaces between C_2F and the anchor loop of C_2E (residues 1475-1514). C_2B and the anchor loop are shown as transparent surfaces.

Appendix Figure S8

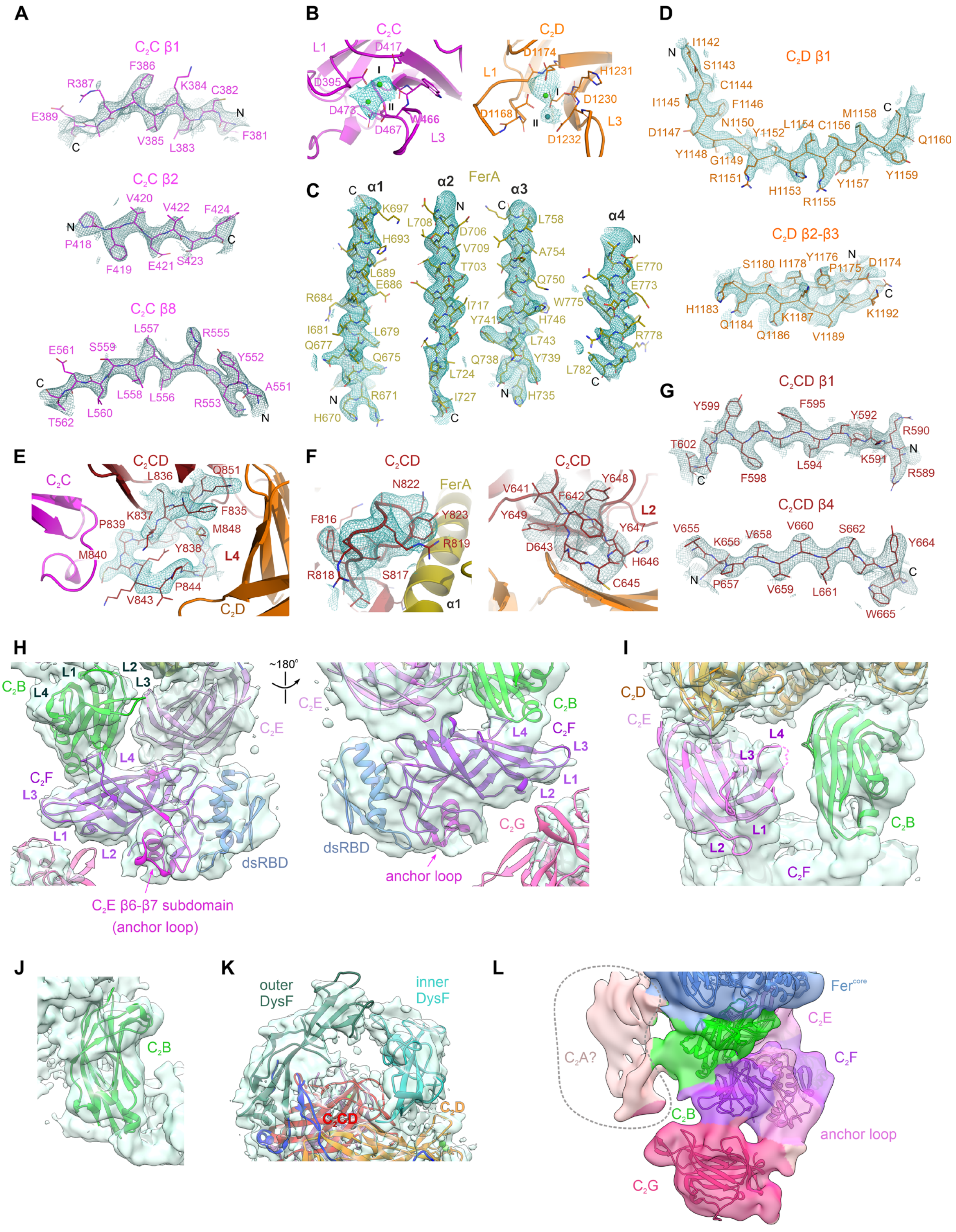

Appendix Figure S8. Snapshots of the lipid-free dysferlin (1-2017) cryo-EM density.

A Snapshots of C_2C's cryo-EM density. C_2C's β-strands are shown as sticks.

B Ca^{2+}-binding sites of dysferlin's C_2C and C_2D domains. The cryo-EM density map is coloured cyan and contoured around the Ca^{2+}-binding sites.

C Cryo-EM density of dysferlin's FerA domain. The FerA α-helices are amphipathic and form a typical up-down-up-down four-helix bundle.

D Cryo-EM densities of C_2D's β-strands.

E Cryo-EM density of C_2CD's L4 loop, bridging the C_2C and C_2D domains.

F Cryo-EM densities of C_2CD's β6-β7 and L2 loops, interacting with the FerA and C_2D domains, respectively.

G Densities of selected C_2CD β-strands.

H-I Modelling of C_2B, C_2E, and C_2F in the closed state cryo-EM map (map M2). The top loops of C_2B and C_2E project onto opposite surfaces of the dysferlin ring, whereas the top loops of C_2F adopt a lateral orientation and face the C_2B domain.

J Side view of C_2B's density with the modelled domain fitted inside the cryo-EM map.

K Cryo-EM densities of the outer and inner DysF motifs of dysferlin. The outer and inner DysF motifs occupy a peripheral location, between C_2CD and C_2D, and share a dynamic contact interface (*i.e.*, not present in all imaged particles).

L Densities of the C-terminal C_2F and C_2G domains. The cryo-EM map (map M3) has been low-pass filtered to 10 Å. The unassigned density element, connected to C_2B, might correspond to the N-terminal C_2A domain, sampling many conformational states in solution. The long linker between C_2A and C_2B is, likely, unstructured. Interestingly, the linker helix (Fig 1D) could not be resolved in the lipid-free dysferlin structure.

Appendix Figure S9

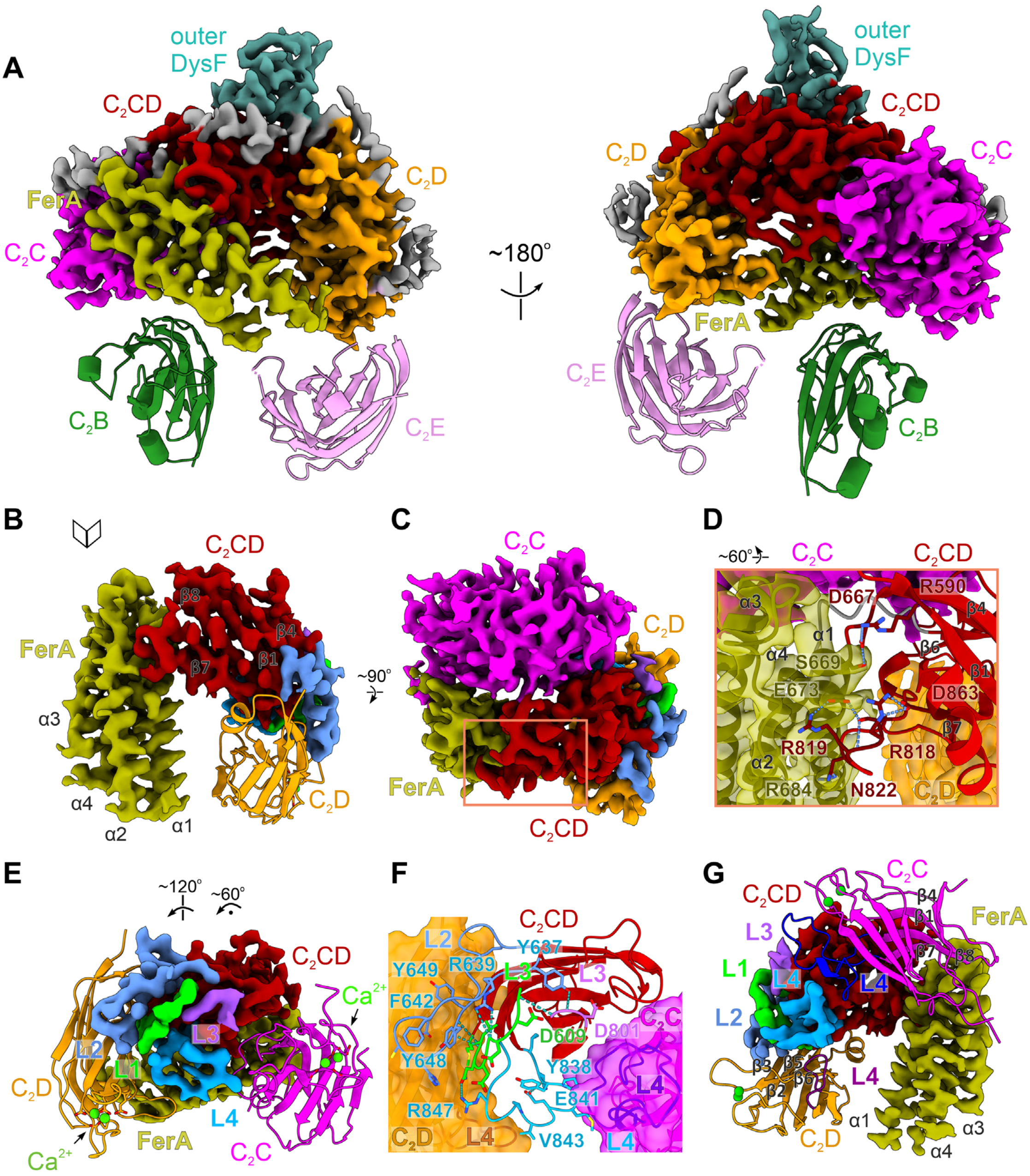

Appendix Figure S9. Structural organization of dysferlin's Fer^{core} module.

A Structure of dysferlin's core module (Fer^{core}) resolved at 2.9 Å resolution. The focused cryo-EM map of the C_2C-C_2D region (map M1), shown in two different orientations, has been sharpened and colour-coded.

B-C Interaction interfaces between C_2CD and its neighbouring FerA, C_2C, and C_2D domains. The sharpened cryo-EM map is coloured according to the modelled domains.

D An insertion in C_2CD's β6-β7 appears to stabilize the conformation of FerA. Dysferlin's domains are depicted in cartoon representation, and the sharpened cryo-EM map is contoured around FerA and C_2D. The key polar interactions between C_2CD's β6-β7 loop and FerA are shown as dashed blue lines.

E C_2CD's top loops engage in multiple contacts with C_2C and C_2D. The L1-L4 loops of C_2CD are coloured green, light blue, purple, and blue, respectively. C_2CD and FerA are shown as cryo-EM maps.

F Selected polar interactions between C_2CD's top loops (L1-L4) and C_2C-C_2D. Polar interactions are shown as dashed blue lines. C_2C and C_2D are displayed as surfaces.

G The large contact interfaces between C_2CD-FerA and the Ca^{2+}-binding C_2C and C_2D domains. C_2CD and FerA are shown as cryo-EM maps, whereas C_2C and C_2D are shown as cartoons.

Appendix Figure S10

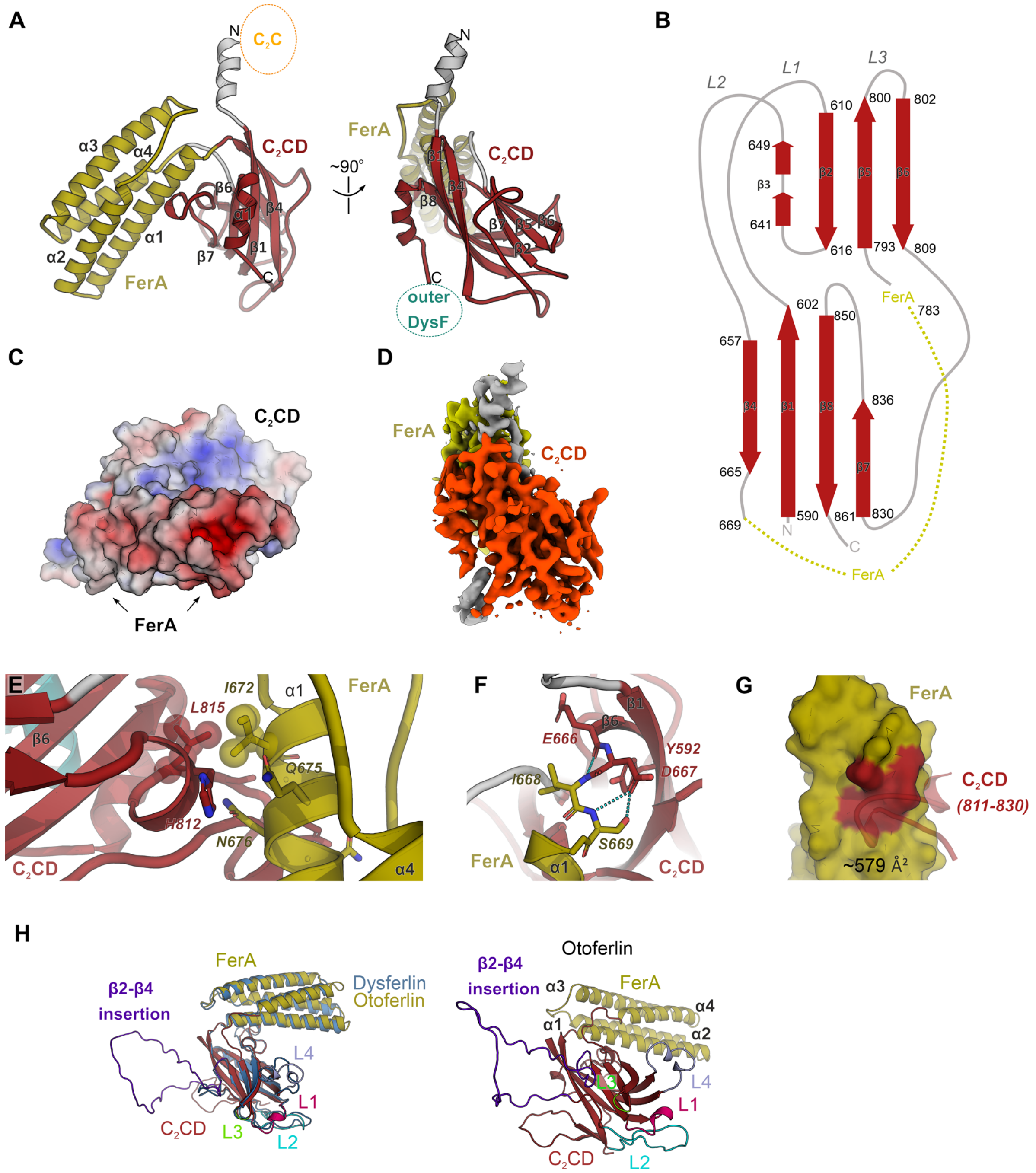

Appendix Figure S10. C_2CD represents a new C_2-like structural domain in ferlins.

A Structure of the C_2CD-FerA module, using human dysferlin as an example. The FerA four-helix bundle inserts between C_2CD's β4-β5 strands.

B Topology diagram of the C_2CD domain. As all other ferlin C_2 domains, C_2CD has a type-II topology: its two β-sheets are formed by the β1-β4-β7-β8 and β2-β3-β5-β6 strands, respectively.

C Surface-charge distribution of the C_2CD-FerA module of dysferlin. The electrostatic potentials are coloured from red (negative, -3 k_BT/e^-) to blue (positive, +3 k_BT/e^-) and mapped onto the solvent-excluded surface of the domains.

D Cryo-EM density of dysferlin's C_2CD-FerA module, oriented and coloured as in A.

E-F Selected contacts between C_2CD and FerA. The polar interactions are represented as dashed lines.

G C_2CD's β6-β7 loop interacts with the FerA domain. FerA's α1 helix is shown as a surface representation, and the C_2CD interacting residues are coloured red.

H A similarly organized C_2CD-FerA module is likely present in all human ferlins. AlphaFold2 prediction of otoferlin's C_2CD-FerA region (right) is depicted side-by-side with its structural superposition to the cryo-EM model of dysferlin's C_2CD-FerA (left). Note that a large insertion between C_2CD's β2-β4 strands is predicted in otoferlin.

Appendix Figure S11

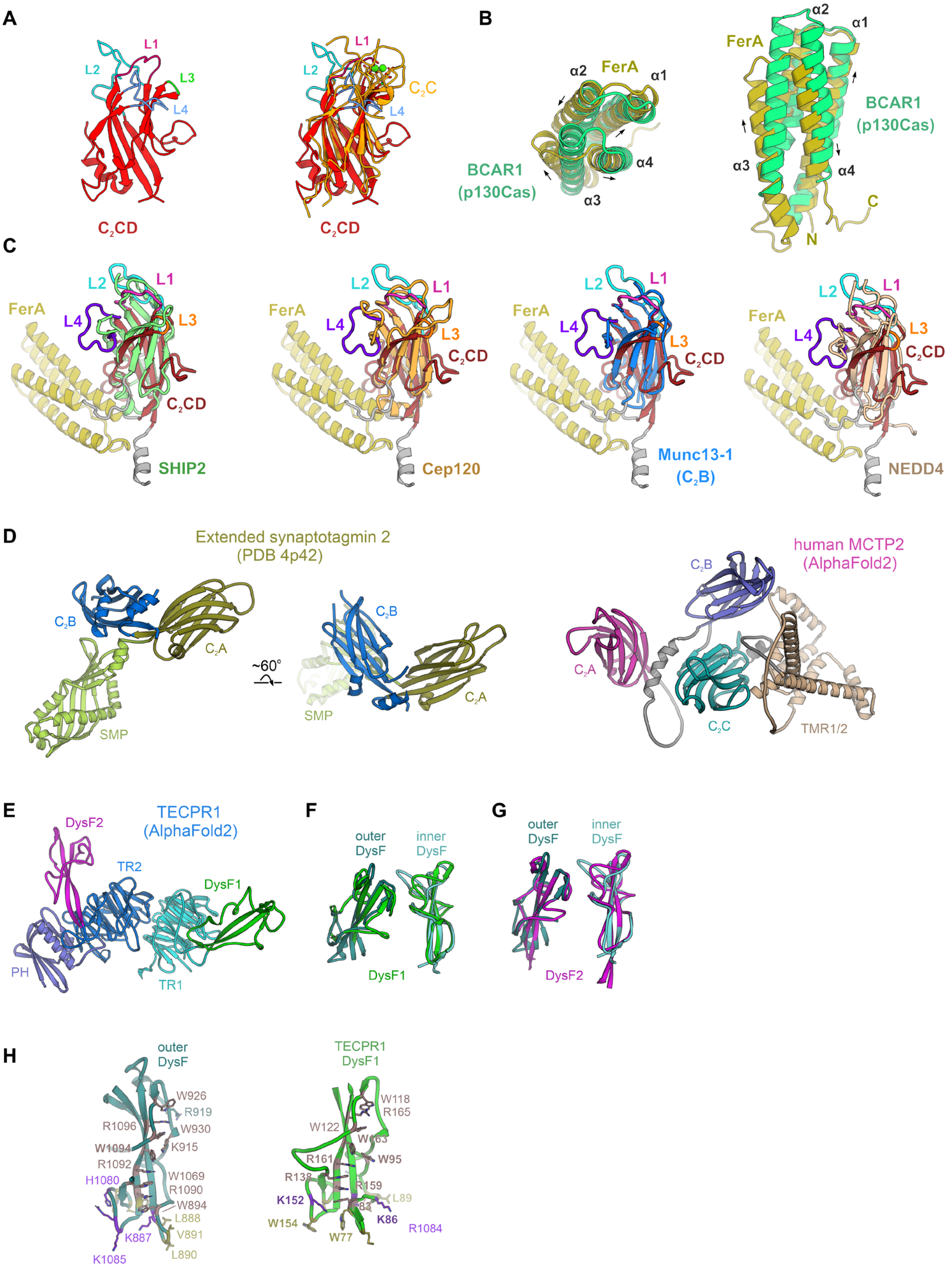

Appendix Figure S11. Structural conservation of ferlin C_2CD, FerA, and DysF motifs.

A Structural superposition of C_2CD and the Ca^{2+}-bound C_2C domain of dysferlin, as an exemplary type-I ferlin. C_2C's Ca^{2+} ions are shown as green spheres.

B Structural similarity between dysferlin's FerA domain and the four-helix bundle of BCAR1 (p130Cas, PDB 3t6g). The direction of the four α-helices is indicated by arrows.

C Alignments between the C_2CD domain of dysferlin and the structurally similar C_2 domains of SHIP2 phosphatase (PDB 5OKM), Cep120 (PDB 6FLJ), Munc13-1 (PDB 5UE8), and NEDD4 (PDB 3M7F). The C_2 domains were aligned using Foldseek (van Kempen, Kim et al., 2024).

D Structures of extended synaptotagmin 2 (ESYT2, PDB 4P42) and human MCTP2 (Multiple C_2 and transmembrane domain-containing protein 2, AlphaFold2 prediction). SMP – synaptotagmin-like mitochondrial-lipid-binding protein domain; TMR1/2 – transmembrane region 1 and 2.

E AlphaFold2 prediction of TECPR1 (Tectonin Beta-Propeller Repeat-containing Protein 1)'s structure. The tectonin repeat β-propellers are coloured blue and cyan, respectively (TR1, TR2). The two spatially separated DysF motifs are coloured green (DysF1) and magenta (DysF2).

F Superposition of dysferlin's outer and inner DysF motifs and the related DysF1 domain of TECPR1, predicted by AlphaFold2 (Jumper et al., 2021).

G Structural superposition between the DysF motifs of dysferlin and TECPR1's DysF2.

H Stabilizing arginine/tryptophan (R/W) stacking interactions are present in both dysferlin/myoferlin's and TECPR1's DysF motifs. In both cases, the R/W stacks (shown as sticks) are mainly observed on one side of the DysF β-sheet (Patel, Harris et al., 2008, Sula, Cole et al., 2014).

Appendix Figure S12

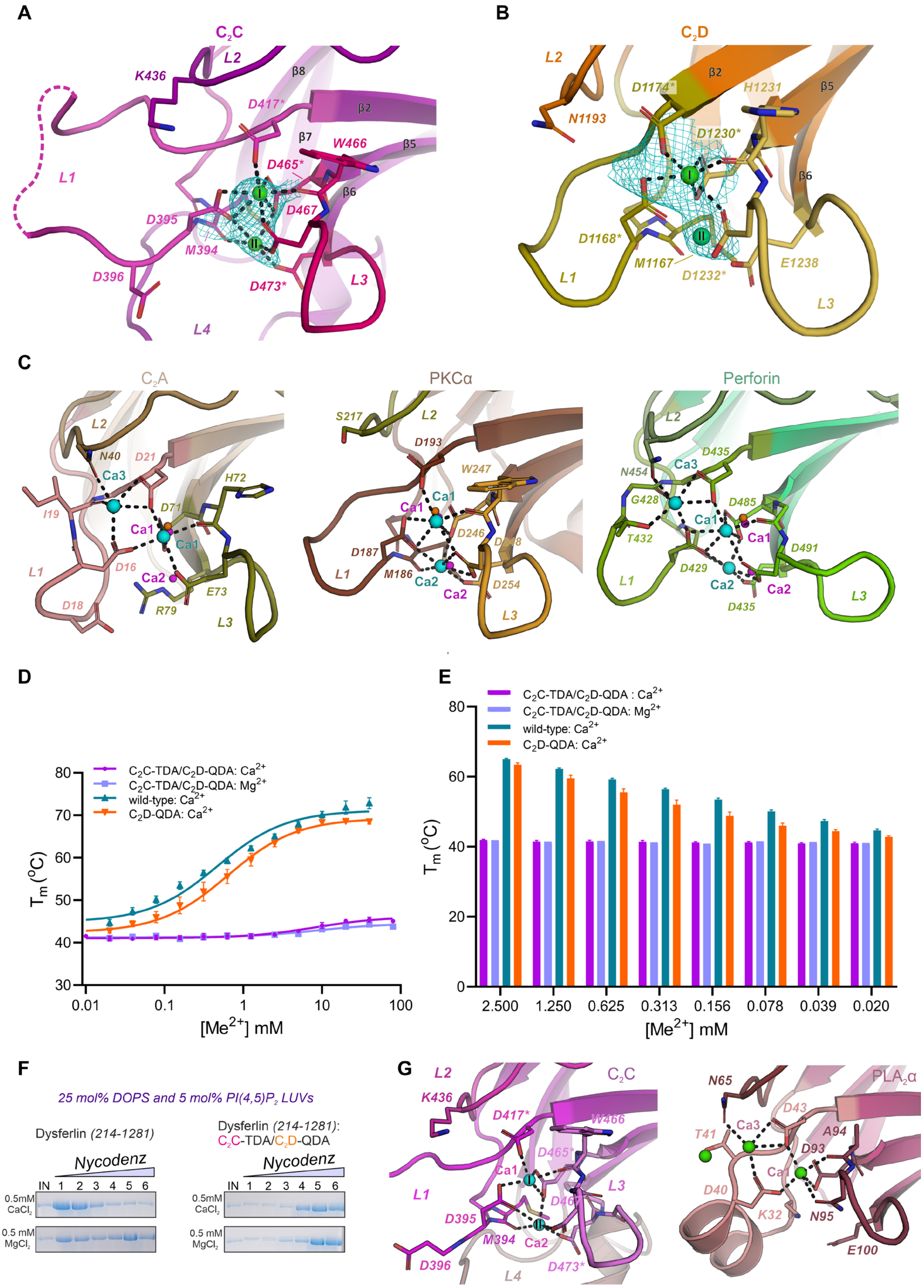

Appendix Figure S12. Ca^{2+}-binding sites in the Fer^{core} module of dysferlin, modelled in the lipid-free state.

A In dysferlin, two Ca^{2+} ions bind to C_2C's Ca^{2+}-binding pocket. The cryo-EM map (cyan mesh) is contoured around the Ca^{2+} ions (green spheres). Residues substituted with alanine (in D-E) are marked with asterisks.

B The Ca^{2+}-binding sites of the C_2D domain. Residues substituted for alanine in D-E are indicated with asterisks.

C Comparison of Ca^{2+} coordination in dysferlin's C_2C and C_2A (PDB 7JOF) with the C_2 domains of PKCα (PDB 1DSY) and perforin (PDB 4Y1T). C_2C's Ca^{2+} ions are shown as magenta spheres, whereas the Ca^{2+} sites of C_2A, PKCα, and perforin are coloured cyan.

D Ca^{2+}-binding activities of the Fer^{core} of dysferlin (residues 214-1281) and of its C_2D-QDA ("quadruple D (aspartate) to A (alanine)") and C_2C-TDA ("triple D (aspartate) to A (alanine)")/C_2D-QDA mutants. In C_2D-QDA, four Ca^{2+}-binding aspartates of C_2D were substituted with alanine (D1168A, D1174A, D1230A, D1232A). In the C_2C-TDA/C_2D-QDA mutant, three additional residues of C_2C were substituted with alanine (D417A, D465A, D473A) (see also Appendix Fig S13A-C). The nanoDSF-based Ca^{2+}-activity measurements were performed in triplicate (n=3) for C_2D-QDA and C_2C-TDA/C_2D-QDA, and five times (n=5) for the wild-type Fer^{core} sample. Error bars represent the standard deviation (s.d.). The melting temperatures (T_m) were plotted as a function of Ca^{2+}/Mg^{2+} concentration, and the $[Ca^{2+}]_{1/2}$ values were estimated by nonlinear regression curve fitting.

E Bar plot of the Ca^{2+}-activity data shown in D. Error bars represent the s.d..

F Ca^{2+}-sensitive liposome binding activity of the wild-type and C_2C-TDA/C_2D-QDA Fer^{core} of dysferlin (residues 214-1281). The liposomes (Large unilamellar vesicles, LUVs) contained 25 mol% DOPS and 5 mol% PI(4,5)P_2 anionic phospholipids. The Nycodenz step gradients (0%/30%/40%) were harvested from the top and analysed by SDS-PAGE. Importantly, disruption of C_2C's and C_2D's Ca^{2+}-binding sites significantly reduced the ability of the Fer^{core} module to interact with lipid membranes.

G Modelled Ca^{2+}-binding sites in dysferlin's C_2C domain and the C_2 domain of cytosolic phospholipase $A_2α$ ($PLA_2α$, PDB 6IEJ). The Ca^{2+} ions are depicted as spheres and the coordinating side chains shown as sticks. Notably, like the C_2C domain of myoferlin (Fig 4F and 4I), $PLA_2α$'s L1 loop features an amphipathic, lipid-binding α-helix (Hirano, Gao et al., 2019).

Appendix Figure S13

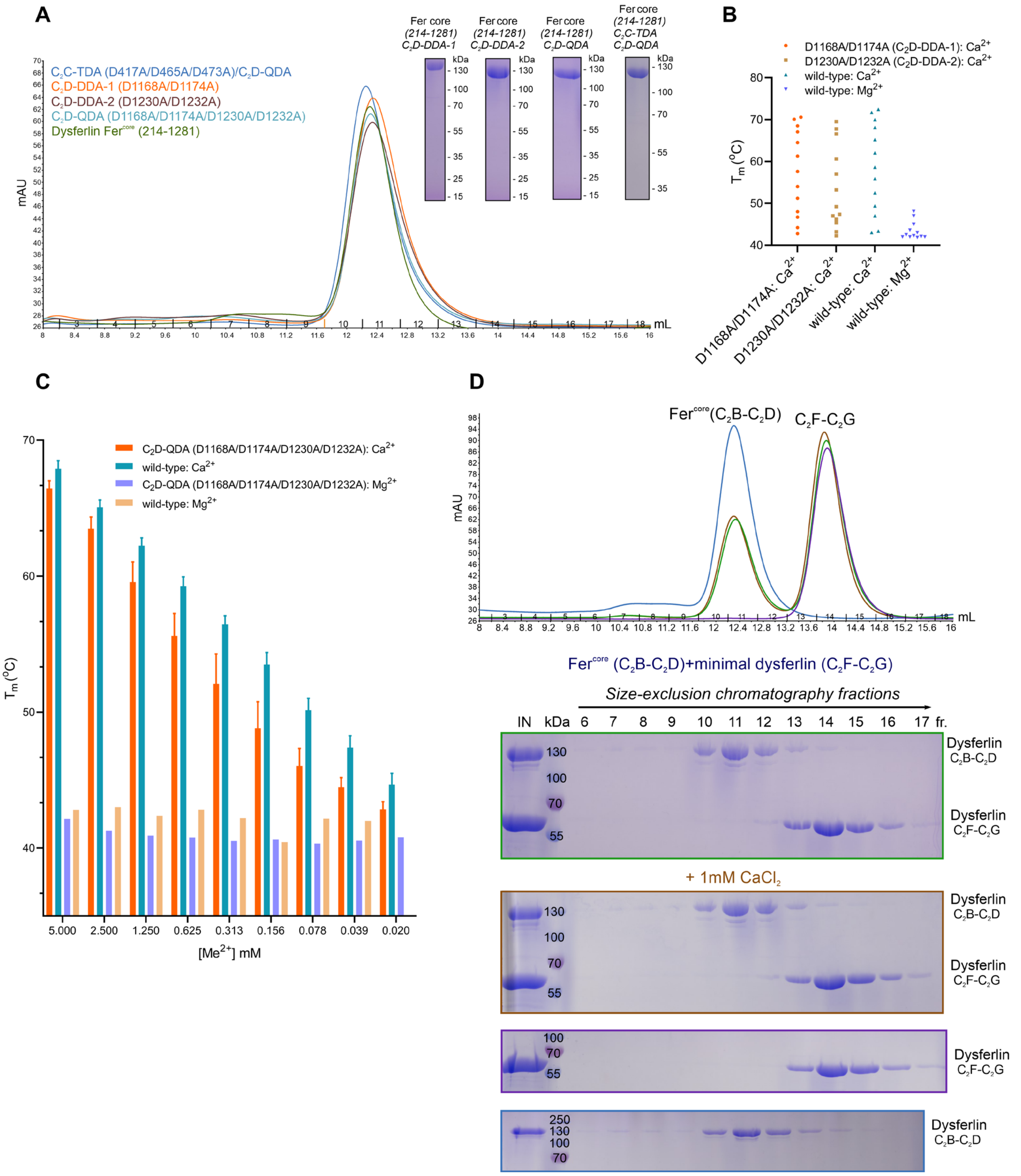

Appendix Figure S13. Biochemical validation of Ca^{2+}-binding sites in dysferlin.

A Size-exclusion chromatography (SEC) profiles of Ca^{2+}-binding mutants in C_2C and C_2D. The purified mutants have retention volumes on Superdex 200 10/300 comparable to the wild-type Fer^{core} (residues 214-1281). Therefore, substitution of two (C_2D-DDA-1: D1168A/D1174A and C_2D-DDA-2: D1230A/D1232A) or four Ca^{2+}-binding aspartates (C_2D-QDA: D1168A/D1174A/D1230A/D1232A) in C_2D does not have a significant impact on the structure of the Fer^{core}. Similarly, combined substitutions in C_2C (C_2C-TDA: D417A/D465A/D473A) and C_2D (C_2D-QDA) preserve the structural integrity of dysferlin's Fer^{core} while abolishing its Ca^{2+}-binding activity (see also Appendix Fig S12D-E). SDS-PAGE gels of the Fer^{core} dysferlin mutants are shown as an inset.

B Ca^{2+}-binding activity of C_2D's DDA ("double D (aspartate) to A (alanine)") substitutions, measured using a nanoDSF assay. The melting temperatures (T_m) of C_2D-DDA-1 (D1168A/D1174A) and C_2D-DDA-2 (D1230A/D1232A) were assessed as a function of [Ca^{2+}] or [Mg^{2+}]. Single nanoDSF T_m estimates are shown (n=1).

C Ca^{2+}-binding activity measurements of the C_2D-QDA (D1168A/D1174A/D1230A/D1232A) Fer^{core} mutant (see also Appendix Fig S12D-E). The estimated T_m values were plotted as a function of [Ca^{2+}] or [Mg^{2+}]. Error bars represent the s.d. (C_2D-QDA: n=3; wild-type: n=5).

D Probing the interaction between the C_2B-C_2D and C_2F-C_2G dysferlin fragments by SEC. The purified samples were mixed in the presence or absence of 1 mM $CaCl_2$ and subjected to SEC on a Superdex 200 10/300 column. The peak fractions were analysed by SDS-PAGE. In these experiments, no complex formation was observed between C_2B-C_2D (Fer^{core}, residues 214-1281) and C_2F-C_2G (minimal dysferlin, residues 1503-1560 and 1613-2056): C_2B-C_2D peaked in fraction 11, whereas the C_2F-C_2G dysferlin fragment peaked in fraction 14. The SEC profiles of the reconstitution experiments are shown at the top.

Appendix Figure S14

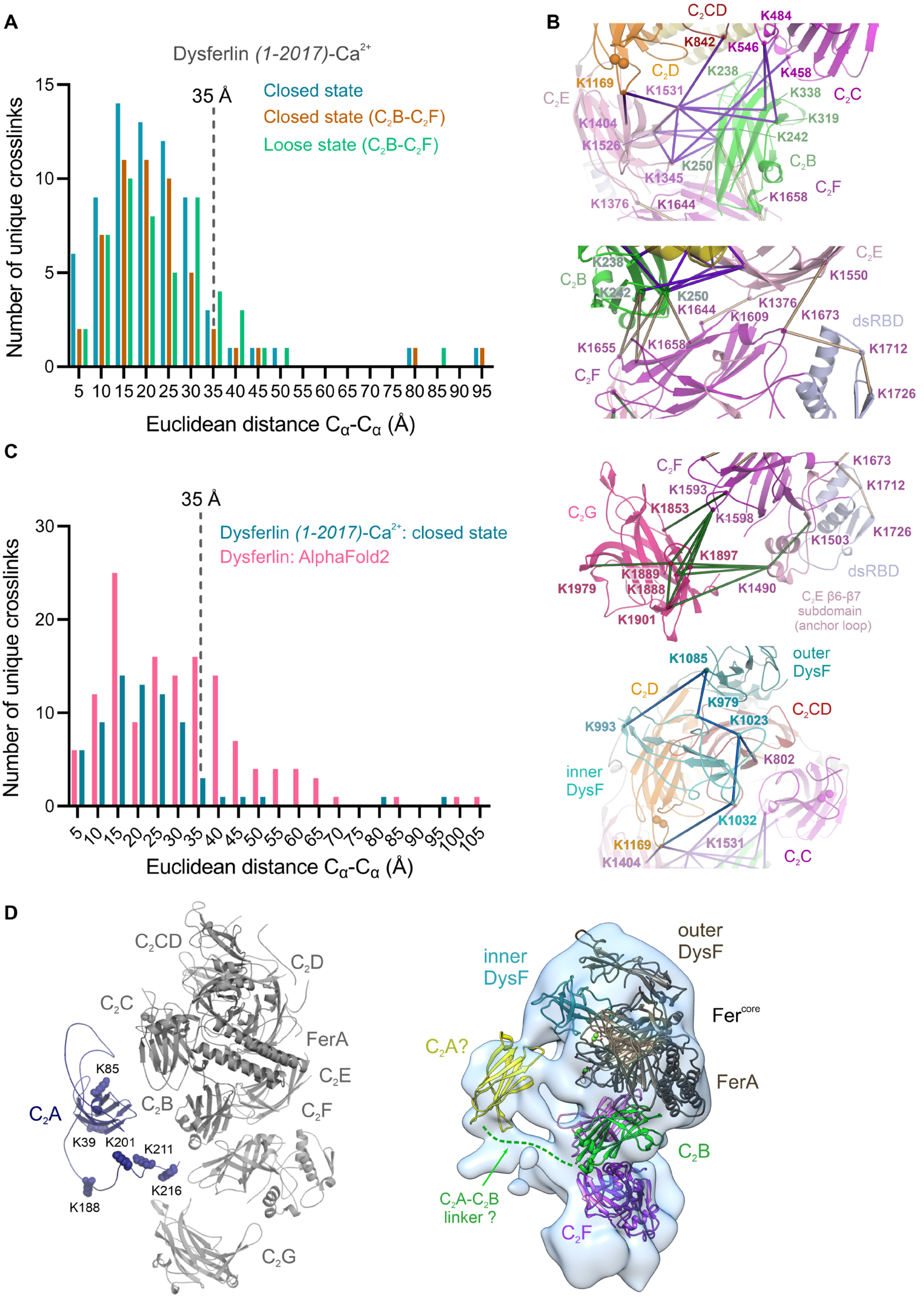

Appendix Figure S14. Chemical-crosslinking mass spectrometry analysis of the Ca^{2+}-bound soluble dysferlin (residues 1-2017).

A Distribution of the Cα-Cα Euclidean distances between crosslinked lysine residues in the closed and loose dysferlin states (Appendix Fig 6A). Dysferlin's chemical crosslinking data indicate that when mapped onto the closed state model, ~93% of unique crosslinks occur between lysine residues less than 35 Å apart (Dataset EV1). Mapping to a loose state model lacking the dynamic C_2G results in ~85% crosslinks satisfying the 35 Å distance threshold, consistent with the possibility that multiple dysferlin conformations coexist in solution.

B Selected chemical crosslinks between dysferlin's structural motifs. Euclidean distances were calculated between the Cα atoms of crosslinked lysine residues in the closed dysferlin state and are depicted as coloured lines.

C Distribution of the crosslink distances in the closed state of soluble dysferlin (residues 1-2017, sDYSF) and a full-length dysferlin AlphaFold2 prediction (model 1, Appendix Fig S15B). Compared to the closed state model, only ~64.9% (37/57) unique crosslinks occur between residues less than 35 Å apart. The crosslink outliers map to the C_2B-C_2F, C_2F-C_2G, and C_2A-C_2B interfaces, indicating that the relative orientation of these domains is not accurately predicted in the AlphaFold2 model.

D BS3 crosslinks involving the flexible C_2A and the C_2A-C_2B linker. The crosslinking data indicate that, although generally flexible, C_2A likely samples a restricted number of conformations near the inner DysF and C_2B. Notably, C_2A could be accommodated by the unassigned density region, which continues from C_2B towards the centre of dysferlin's (and myoferlin's) membrane-binding surface.

Appendix Figure S15

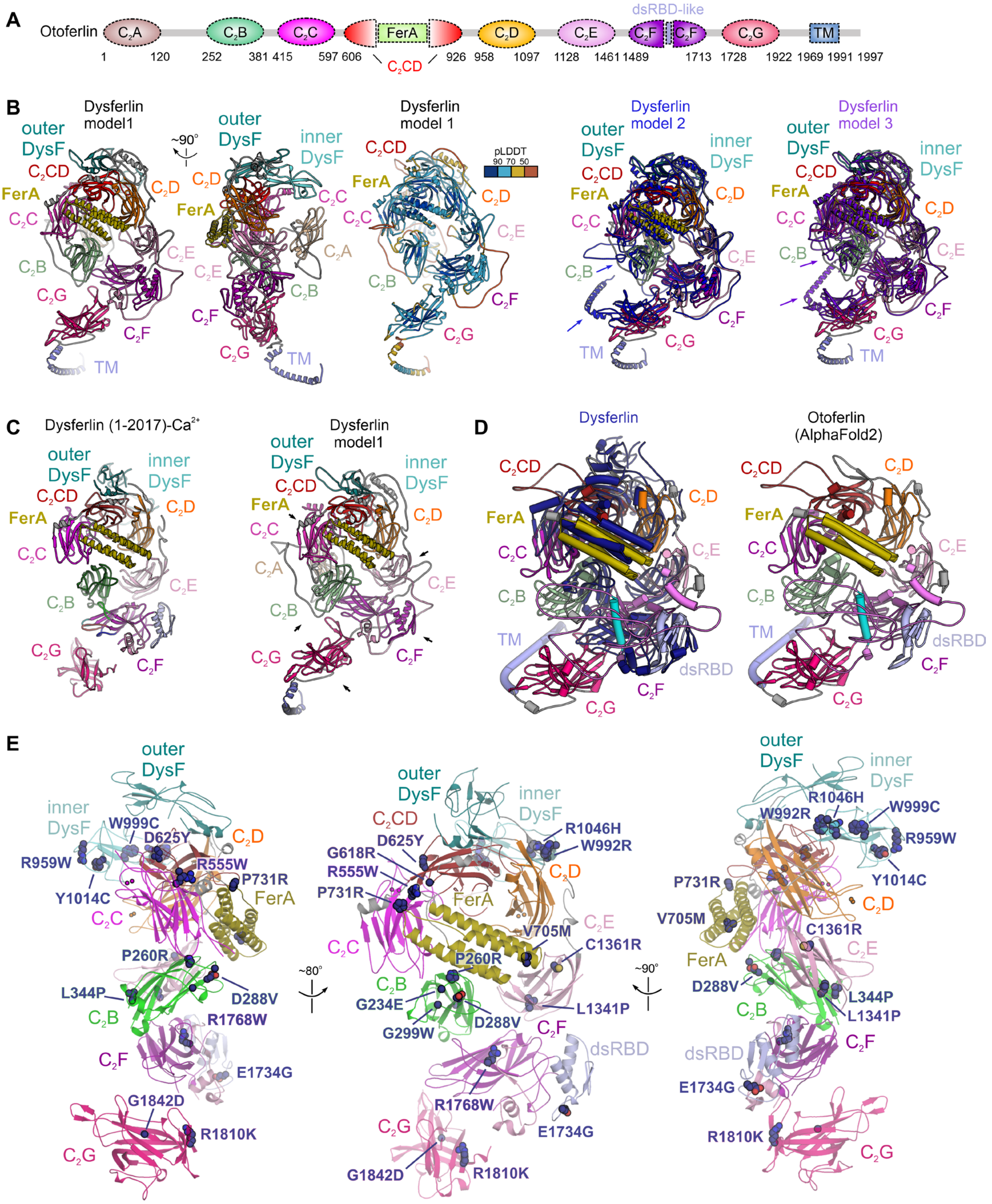

Appendix Figure S15. Common organization principles of human ferlins.

A AlphaFold2-based domain composition schematic of human otoferlin (Jumper, Evans et al., 2021).

B AlphaFold2 models of human dysferlin. While the Fer^{core} module is similar in these predictions, C_2B, C_2E, C_2F, C_2G, and the transmembrane region (TM) adopt alternative poses (marked by arrows), consistent with them being structurally dynamic. The long linkers connecting the C_2A-C_2B and C_2E-C_2F are likely unstructured, with pLDDT (predicted local distance difference test) scores <50.

C Comparison between dysferlin's closed state (Appendix Fig S6A) and an AlphaFold2 prediction (model 1) of full-length dysferlin. Note the significantly different poses of the C_2B, C_2E, C_2F, and C_2G domains (indicated by arrows, r.m.s.d.=4.85 over 1156 atoms). Minor differences are observed for the FerA domain. Conversely, the Fer^{core} module (except the inner DysF motif) is similarly organized in the dysferlin cryo-EM structure and AlphaFold2 models (r.m.s.d.=1.79 over 618 atoms).

D Comparison between dysferlin's cryo-EM model and an AlphaFold2 prediction of human otoferlin. Otoferlin lacks the DysF motifs and the C_2E anchor loop (magenta) is extended. In addition, C_2B and C_2E-C_2F are arranged in a circular conformation – a configuration that appears reminiscent of the lipid-bound myoferlin structures (Fig 1C-D and Fig EV2).

E Pathogenic mutations mapped onto the lipid-free dysferlin structure. Pathogenic and likely pathogenic dysferlin substitutions were extracted from the ClinVar database (Landrum, Chitipiralla et al., 2020) and manually curated. Some dysferlin substitutions appear to cluster at tertiary interfaces, such as between C_2C and C_2CD (*e.g.*, R555W and D625Y).

Appendix Table S1. Data collection, refinement, and validation statistics for the lipid-bound myoferlin (1-1997): 25 mol% DOPS and 5 mol% PI(4,5)P_2 nanodisc complex (PDB 9H6X/EMD-51902).

	Map M1 (consensus map)		Map M2	Map M3 (overall map)
**Data collection and processing**	Dataset 1	Dataset 2		
Electron gun	X-FEG	X-FEG		
Detector	Falcon4i	Falcon4i		
Magnification	165,000	165,000		
Energy filter slit width (eV)	10	10		
Voltage (kV)	300.0	300.0		
Dose rate ($e^-/A^2/s$)	12.53	12.41		
Electron exposure on sample (e^-/A^2)	39.595	39.216		
Target defocus range (μm)	0.8-2.0	0.8-2.3		
Calibrated pixel size (Å)	0.72	0.72		
Symmetry imposed	C1	C1		
Collected movies (no.)	8,660	22,379		
Initial particle images (no.)	1,893,590	5,758,253		
Final particle images (no.)	1,585,485		1,101,310	233,637
Map resolution at FSC=0.143 (Å)	2.39		2.46	2.56
**Refinement**				
Initial model used	AlphaFold2			AlphaFold2
Model resolution (Å)	2.5			2.8
Model resolution at FSC=0.5 (Å)	2.65			2.86
Map sharpening B factor ($Å^2$)*	-90.7			-85.5
Model composition				
Non-hydrogen atoms	12,913			13,813
Protein residues	1,585			1,693
Ligands	2x PS			2x PS
Waters	-			-
Ions	10x Ca^{2+}			10x Ca^{2+}
B factors ($Å^2$)				
Protein (min/max/mean)	72.29/349.12/163.66			54.49/367.34/152.37
Ligand (min/max/mean)	98.4/316.96/163.64			88.25/298.69/158.12
Water	-			-
R.m.s. deviations				
Bond lengths (Å)	0.005			0.004
Bond angles (°)	0.950			0.840
**Validation**				
Molprobity score	1.24			1.20
Clashscore	1.79			2.08
Rotamer outliers (%)	0.85			0.86
Cβ outliers (%)	-			-
CaBLAM outliers (%)	2.75			2.16
Ramachandran plot				
Favored (%)	95.61			96.55
Allowed (%)	4.32			3.45
Disallowed (%)	0.06			-

*Automatically determined B factor; the maps were sharpened using LocScale and DeepEMhancer.

Appendix Table S2. Data collection, refinement, and validation statistics for the lipid-bound myoferlin (1-1997): 25 mol% DOPS, 5 mol% PI(4,5)P_2 and 5 mol% cholesterol nanodisc complex (PDB 9QLF/EMD-53226).

	Map M4 A (consensus map)		Map M4 C	Map M4 B (overall map)
**Data collection and processing**	Dataset 1	Dataset 2		
Electron gun	X-FEG	X-FEG		
Detector	Falcon4i	Falcon4i		
Magnification	165,000	165,000		
Energy filter slit width (eV)	10	10		
Voltage (kV)	300.0	300.0		
Dose rate ($e^-/A^2/s$)	10.99	15.82		
Electron exposure on sample (e^-/A^2)	38.025	38.917		
Target defocus range (μm)	0.8-2.0	0.8-2.0		
Calibrated pixel size (Å)	0.72	0.72		
Symmetry imposed	C1	C1		
Collected movies (no.)	8,825	12,511		
Initial particle images (no.)	2,445,037	2,594,384		
Final particle images (no.)	882,916		319,420	94,183
Map resolution at FSC=0.143 (Å)	2.58		2.65	3.27
**Refinement**				
Initial model used	AlphaFold2		AlphaFold2	AlphaFold2
Model resolution (Å)	2.8		3.0	3.4
Model resolution at FSC=0.5 (Å)	2.90		2.89	3.54
Map sharpening B factor ($Å^2$)*	-100.7		-95.4	-82.0
Model composition				
Non-hydrogen atoms	12,901		13,765	13,765
Protein residues	1,583		1,687	1,687
Ligands	2x PS		2x PS	2x PS
Waters	-		-	-
Ions	10x Ca^{2+}		10x Ca^{2+}	10x Ca^{2+}
B factors ($Å^2$)				
Protein (min/max/mean)	52.38/380.56/139.06		42.46/375.33/138.82	46.09/395.50/145.05
Ligand (min/max/mean)	89.96/262.07/154.90		74.75/265.83/137.53	99.11/285.69/159.16
Water	-		-	-
R.m.s. deviations				
Bond lengths (Å)	0.005		0.005	0.005
Bond angles (°)	0.968		0.897	0.944
**Validation**				
Molprobity score	1.43		1.24	1.49
Clashscore	2.53		1.76	2.34
Rotamer outliers (%)	1.62		1.33	1.79
Cβ outliers (%)	-		-	-
CaBLAM outliers (%)	2.12		1.69	1.75
Ramachandran plot				
Favored (%)	96.5		96.65	95.88
Allowed (%)	3.44		3.35	4.12
Disallowed (%)	0.06		-	-

*Automatically determined B factor

Appendix Table S3. Data collection, refinement, and validation statistics for the lipid-bound myoferlin (1-1997): 15 mol% DOPS and 5 mol% cholesterol nanodisc complex (9QKV/EMD-53222).

	Map M5 (consensus map)	Map M6 (overall map)
**Data collection and processing**		
Electron gun	X-FEG	
Detector	Falcon4i	
Magnification	165,000	
Energy filter slit width (eV)	10	
Voltage (kV)	300.0	
Dose rate ($e^-/A^2/s$)	11.55	
Electron exposure on sample (e^-/A^2)	39.848	
Target defocus range (µm)	0.8-2.0	
Calibrated pixel size (Å)	0.72	
Symmetry imposed	C1	
Collected movies (no.)	9,621	
Initial particle images (no.)	2,917,286	
Final particle images (no.)	277,729	38,088
Map resolution at FSC=0.143 (Å)	2.74	3.29
**Refinement**		
Initial model used	AlphaFold2	AlphaFold2
Model resolution (Å)	3.0	3.4
Model resolution at FSC=0.5 (Å)	3.1	3.6
Map sharpening B factor ($Å^2$)*	-97.7	-63.9
Model composition		
Non-hydrogen atoms	13,766	13,766
Protein residues	1,688	1,688
Ligands	2x PS	2x PS
Waters	-	-
Ions	10x Ca^{2+}	10x Ca^{2+}
B factors ($Å^2$)		
Protein (min/max/mean)	32.54/361.56/122.53	56.5/364.23/149.37
Ligand (min/max/mean)	60.97/252.15/114.85	97.92/313.94/165.12
Water	-	
R.m.s. deviations		
Bond lengths (Å)	0.005	0.005
Bond angles (°)	0.970	1.018
**Validation**		
Molprobity score	1.24	1.38
Clashscore	2.01	2.60
Rotamer outliers (%)	0.73	1.33
Cβ outliers (%)	-	0.13
CaBLAM outliers (%)	1.69	2.59
Ramachandran plot		
Favored (%)	96.06	96.36
Allowed (%)	3.88	3.58
Disallowed (%)	0.06	0.06

*Automatically determined B factor

Appendix Table S4. Data collection, refinement, and validation statistics for the lipid-bound myoferlin (1-1997): 15 mol% DOPS and 2 mol% PI(4,5)P_2 nanodisc (9QLE/EMD-53225).

	Map M7 (consensus map)	Map M8 (overall map)
**Data collection and processing**		
Electron gun	X-FEG	
Detector	Falcon4i	
Magnification	165,000	
Energy filter slit width (eV)	10	
Voltage (kV)	300.0	
Dose rate ($e^-/A^2/s$)	11.53	
Electron exposure on sample (e^-/A^2)	39.894	
Target defocus range (µm)	1.1-2.0	
Calibrated pixel size (Å)	0.72	
Symmetry imposed	C1	
Collected movies (no.)	7,014	
Initial particle images (no.)	2,484,379	
Final particle images (no.)	583,145	28,855
Map resolution at FSC=0.143 (Å)	2.79	3.43
**Refinement**		
Initial model used	AlphaFold2	AlphaFold2
Model resolution (Å)	3.0	3.6
Model resolution at FSC=0.5 (Å)	3.12	3.82
Map sharpening B factor (Å^2)*	-107.9	-66.9
Model composition		
Non-hydrogen atoms	13,823	13,823
Protein residues	1,695	1,695
Ligands	2x PS	2x PS
Waters	-	-
Ions	10x Ca^{2+}	10x Ca^{2+}
B factors (Å^2)		
Protein (min/max/mean)	46.58/437.76/135.50	80.52/339.96/142.26
Ligand (min/max/mean)	87.60/224.29/132.75	112.33/233.83/147.31
Water	-	-
R.m.s. deviations		
Bond lengths (Å)	0.004	0.005
Bond angles (°)	0.965	1.003
**Validation**		
Molprobity score	1.26	1.43
Clashscore	1.71	2.62
Rotamer outliers (%)	1.52	1.26
Cβ outliers (%)	-	-
CaBLAM outliers (%)	1.86	2.45
Ramachandran plot		
Favored (%)	96.79	95.66
Allowed (%)	3.21	4.28
Disallowed (%)	-	0.06

*Automatically determined B factor

Appendix Table S5. Data collection, refinement, and validation statistics for the lipid-free soluble myoferlin (residues 1-1997, PDB 9QLN/EMD-53229).

	Map M9 (consensus map)		Map M10 (Fer^{core})	Map M11 (overall map)
**Data collection and processing**	Dataset 1	Dataset 2		
Electron gun	X-FEG	X-FEG		
Detector	Falcon4i	Falcon4i		
Magnification	165,000	165,000		
Energy filter slit width (eV)	10	10		
Voltage (kV)	300.0	300.0		
Dose rate ($e^-/A^2/s$)	11.67	10.8		
Electron exposure on sample (e^-/A^2)	39.911	39.852		
Target defocus range (μm)	1.1-2.3	0.8-2.0		
Calibrated pixel size (Å)	0.72	0.72		
Symmetry imposed	C1	C1		
Collected movies (no.)	10,354	8,669		
Initial particle images (no.)	6,088,884	3,020,482		
Final particle images (no.)	102,339		298,755	18,157
Map resolution at FSC=0.143 (Å)	3.21		2.81	8.5
**Refinement**				
Initial model used	AlphaFold2			
Model resolution (Å)	3.4			
Model resolution at FSC=0.5 (Å)	3.73			
Map sharpening B factor ($Å^2$)*	-91.9			
Model composition				
Non-hydrogen atoms	10,833			
Protein residues	1,340			
Ligands	-			
Waters	-			
Ions	6x Ca^{2+}			
B factors ($Å^2$)				
Protein (min/max/mean)	29.97/419.31/167.9			
Ligand (min/max/mean)	95.77/336.41/180.97			
Water	-			-
R.m.s. deviations				
Bond lengths (Å)	0.005			
Bond angles (°)	1.002			
**Validation**				
Molprobity score	1.51			
Clashscore	3.06			
Rotamer outliers (%)	1.34			
Cβ outliers (%)	-			
CaBLAM outliers (%)	2.74			
Ramachandran plot				
Favored (%)	95.48			
Allowed (%)	4.45			
Disallowed (%)	0.08			

*Automatically determined B factor; the maps were sharpened using LocScale.

Appendix Table S6. Data collection, refinement, and validation statistics for the lipid-free soluble dysferlin (residues 1-2017, PDB 9QLS, EMD-53233).

	Map M1 (Fer^{core} map)		Map M2 (closed state)	Map M3 (overall map)
**Data collection and processing**	Dataset 1	Dataset 2		
Electron gun	X-FEG	XFEG		
Detector	Falcon4i	Falcon4i		
Magnification	165,000	165,000		
Energy filter slit width (eV)	15	10		
Voltage (kV)	300.0	300.0		
Dose rate ($e^-/Å^2/s$)	13.35	13.59		
Electron exposure on sample ($e^-/Å^2$)	40.184	40.227		
Target defocus range (µm)	0.8-2.3	0.8-2.3		
Calibrated pixel size (Å)	0.72	0.72		
Symmetry imposed	C1	C1		
Collected movies (no.)	4,582	4,727		
Initial particle images (no.)	1,660,317	1,201,065		
Final particle images (no.)	230,540		29,793	11,477
Map resolution at FSC=0.143 (Å)	2.92		3.54	4.76
**Refinement**				
Initial model used			AlphaFold2	
Model resolution (Å)			3.6	
Model resolution at FSC=0.5 (Å)			3.81	
Map sharpening B factor ($Å^2$)*			-56.9	
Model composition				
Non-hydrogen atoms			11,100	
Protein residues			1,370	
Ligands			-	
Waters			-	
Ions			6x Ca^{2+}	
B factors ($Å^2$)				
Protein (min/max/mean)			45.60/370.52/173.98	
Ligand (min/max/mean)			75.34/297.09/150.49	
Water			-	
R.m.s. deviations				
Bond lengths (Å)			0.004	
Bond angles (°)			0.982	
**Validation**				
Molprobity score			1.54	
Clashscore			2.68	
Rotamer outliers (%)			1.57	
Cβ outliers (%)			-	
CaBLAM outliers (%)			2.19	
Ramachandran plot				
Favored (%)			95.17	
Allowed (%)			4.75	
Disallowed (%)			0.07	

*Automatically determined B factor; the maps were sharpened using LocScale.

Appendix Table S7. PCR primers used in this study.

Primer	Sequence (5'-3')	Construct
CC10_REV	GTCTTCCAACTTGGGGTTCATGTTAG (RH)	pCC17, pCC18
CC11_FWD	TGAGGTACCAAGCTTGTCGAGAAGTAC (RH)	pCC17, pCC18
CC14_FWD	TCTCCTCCTTCATCCCCTCACGA (RH)	pCC19
CC15_REV	TCCAGGACCTTGGAACAGCACCTCCAG (RH)	pCC19
CC34_REV	CATGCCACCGGATCCGCCACCTG (RH)	pCC26
CC35_FWD	AGCGACAAGCCCCAAGACTTCCAG (RH)	pCC26
CC70_FWD	GGAAGCGGAAACGGAAACGCCCAAGGTCCACAGGAATGCT (RH)	pCC48
CC69_REV	GAAGTCAGACAAACCCTCGAAGGCTTCC (RH)	pCC48
CC104_FWD	TACTTCCAATCCAATGCAATGCTGCGAGTGATTGTGGAATCTG (LIC)	pCC68
CC105_REV	TTATCCACTTCCAATGTTATTACACATTTGGCTTTACAATCTTCATTGAC (LIC)	pCC68
CC117_REV	CTTCTCGCGCTGGATCAGTTCGAAAGAG (+CC11_FWD, RH)	pCC75
CC118_REV	GTCCAGCTTGGGGTTCATGTTGGGTTC (RH)	pCC76
CC119_FWD	TAATAACATTGGAAGTGGATAACCGGTCCGA (RH)	pCC76
CC121_FWD	CGTCGCCCCTTTCGTGGAAGTCTCTTTCGCTG (RH)	pCC95
CC122_REV	AGGTTCTTCTTGTTAGACTCGAAGCCGAAG (RH)	pCC95
CC123_FWD	CAACGCCATCGTCGCCACCACTTACCTGTC (RH)	pCC94
CC125_REV	TGAGTCAACCTGTCCCAGGCGATGATCCTG (RH)	pCC94
CC126_FWD	CTATGGCCAAGGACTCTTTCAGCGCCCCATACGC (RH)	pCC84
CC127_REV	CAGCCAAGTCGCGGGCTTGGTAC (RH)	pCC84
CC128_FWD	GCTGTACGCCCACGCCACCTACGGCGCCGAC (RH)	pCC85, pCC90
CC129_REV	TCGACCACGATTGAAGGTGGCTGCTCG (RH)	pCC85, pCC90

RH – "around-the-horn" PCR (using 5'-phosphorylated primers); LIC – ligation-independent cloning

References

Hirano Y, Gao YG, Stephenson DJ, Vu NT, Malinina L, Simanshu DK, Chalfant CE, Patel DJ, Brown RE (2019) Structural basis of phosphatidylcholine recognition by the C2-domain of cytosolic phospholipase A2alpha. Elife 8

Jumper J, Evans R, Pritzel A, Green T, Figurnov M, Ronneberger O, Tunyasuvunakool K, Bates R, Zidek A, Potapenko A, Bridgland A, Meyer C, Kohl SAA, Ballard AJ, Cowie A, Romera-Paredes B, Nikolov S, Jain R, Adler J, Back T et al. (2021) Highly accurate protein structure prediction with AlphaFold. Nature 596: 583-589

Landrum MJ, Chitipiralla S, Brown GR, Chen C, Gu B, Hart J, Hoffman D, Jang W, Kaur K, Liu C, Lyoshin V, Maddipatla Z, Maiti R, Mitchell J, O'Leary N, Riley GR, Shi W, Zhou G, Schneider V, Maglott D et al. (2020) ClinVar: improvements to accessing data. Nucleic Acids Res 48: D835-D844

Patel P, Harris R, Geddes SM, Strehle EM, Watson JD, Bashir R, Bushby K, Driscoll PC, Keep NH (2008) Solution structure of the inner DysF domain of myoferlin and implications for limb girdle muscular dystrophy type 2b. J Mol Biol 379: 981-90

Sula A, Cole AR, Yeats C, Orengo C, Keep NH (2014) Crystal structures of the human Dysferlin inner DysF domain. BMC Struct Biol 14: 3

van Kempen M, Kim SS, Tumescheit C, Mirdita M, Lee J, Gilchrist CLM, Soding J, Steinegger M (2024) Fast and accurate protein structure search with Foldseek. Nat Biotechnol 42: 243-246
