# Supplementary material for: Structural insights into lipid membrane binding by human ferlins
Source: EMBO J. 2025 May 28;44(14):3926–58. doi: 10.1038/s44318-025-00463-8 (PMC12264198; doi:10.1038/s44318-025-00463-8)
Supplement: Supplementary file 4 — Movie EV1 [file 44318_2025_463_MOESM4_ESM.zip › Movie EV1/Movie EV1 Legend.docx]

**Movie EV1. Overall cryo-EM map of lipid (nanodisc)-bound soluble myoferlin (residues 1-1997).**

The overall cryo-EM map of myoferlin (residues 1-1997) bound to an MSP2N2 nanodisc (comprising 25 mol% DOPS and 5 mol% PI(4,5)P_2_) has been coloured after the modelled domains. To better visualize the ordered nanodisc density, the map has been superimposed on a low-pass filtered (to 8 Å) cryo-EM density map (grey).
