# Supplementary material for: Structural insights into lipid membrane binding by human ferlins
Source: EMBO J. 2025 May 28;44(14):3926–58. doi: 10.1038/s44318-025-00463-8 (PMC12264198; doi:10.1038/s44318-025-00463-8)
Supplement: Supplementary file 5 — Movie EV2 [file 44318_2025_463_MOESM5_ESM.zip › Movie EV2/Movie EV2 Legend.docx]

**Movie EV2. Conformational space of myoferlin particles in the lipid (nanodisc)-bound state.**

The 3D variability analysis of myoferlin particles was performed in cryoSPARC v. 4.5. One eigenvector (component) of particles’ 3D variability (filtered to 6 Å) is represented as a series of 20 frames.
