# Supplementary material for: Structural insights into lipid membrane binding by human ferlins
Source: EMBO J. 2025 May 28;44(14):3926–58. doi: 10.1038/s44318-025-00463-8 (PMC12264198; doi:10.1038/s44318-025-00463-8)
Supplement: Supplementary file 6 — Movie EV3 [file 44318_2025_463_MOESM6_ESM.zip › Movie EV3/Movie EV3 Legend.docx]

**Movie EV3. Overall cryo-EM map of human dysferlin (residues 1-2017) in the lipid-free state.**

The cryo-EM density map has been locally scaled, low-pass filtered to 5 Å and colour-coded according to dysferlin’s domains.
