# Supplementary material for: Structural insights into lipid membrane binding by human ferlins
Source: EMBO J. 2025 May 28;44(14):3926–58. doi: 10.1038/s44318-025-00463-8 (PMC12264198; doi:10.1038/s44318-025-00463-8)
Supplement: Supplementary file 7 — Movie EV4 [file 44318_2025_463_MOESM7_ESM.zip › Movie EV4/Movie EV4 Legend.docx]

**Movie EV4. Structural superposition of the lipid-free and lipid (nanodisc)-bound states of myoferlin.**

The two structures were aligned based on the more rigid Fer^core^ module and the individual domains of myoferlin were coloured as in Fig 1 and Fig 7.
