# Supplementary material for: Structural insights into lipid membrane binding by human ferlins
Source: EMBO J. 2025 May 28;44(14):3926–58. doi: 10.1038/s44318-025-00463-8 (PMC12264198; doi:10.1038/s44318-025-00463-8)
Supplement: Supplementary file 8 — Movie EV5 [file 44318_2025_463_MOESM8_ESM.zip › Movie EV5/Movie EV5 Legend.docx]

**Movie EV5. The modelled conformational transition between the lipid-free and membrane-bound states of myoferlin.**

The two states of myoferlin were superimposed based on the Fer^core^ region and the trajectories of domain movement between the two structures (morphing) were estimated in ChimeraX.
