# Supplementary material for: Structural insights into lipid membrane binding by human ferlins
Source: EMBO J. 2025 May 28;44(14):3926–58. doi: 10.1038/s44318-025-00463-8 (PMC12264198; doi:10.1038/s44318-025-00463-8)

General comments

The 2D class images shown in Fig EV3 and Appendix Fig S2, S4 and S5 are 2D averages of the same protein sample (myoferlin (1-1997)), imaged in independent experiments - lipid-free (Fig EV3) or in complex with MSP2N2-based nanodiscs. For illustrative purposes, we have chosen to show almost all calculated high-resolution 2D classes of the imaged samples, even highly similar ones, so that the reader can have a more complete overview of the distribution of particle views in our datasets.

Images of 2D averages of myoferlin (1-1997), shown in EV3 and Appendix Fig S2, S4 and S5 were generated as follows: 1. 2D classification in cryoSPARC; 2. Selection of high-resolution 2D classes in cryoSPARC; 3. Generation of publication-grade images of the selected 2D classes using the cryosparc-tools Python package and the dedicated Python script (https://tools.cryosparc.com/examples/hi-res-2d-classes.html).

Fig EV3B: Lipid-free myoferlin (1-1997)

2D classification job (job id: J5654, cryoSPARC v.4.5.1), 30 2D classes, 81,269 particles

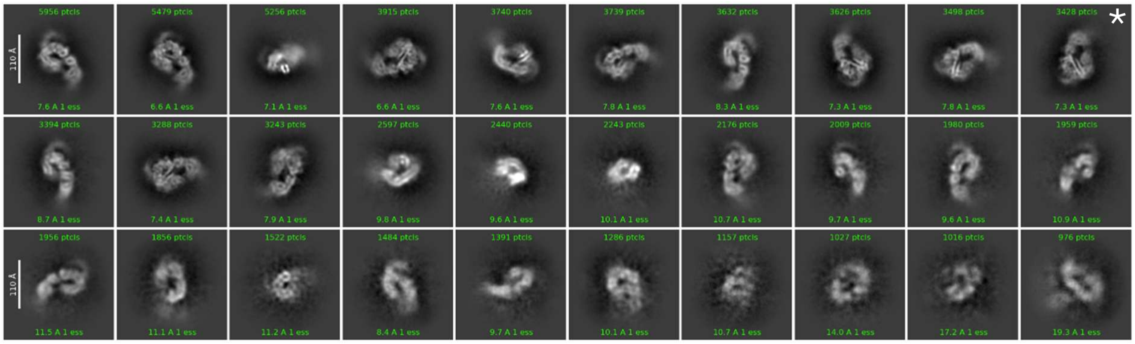

*3,428 particles

Selected 2D classes: 15

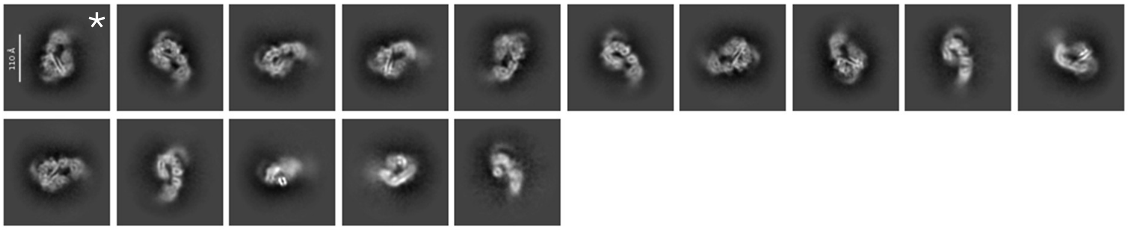

Omitted 2D classes: 15

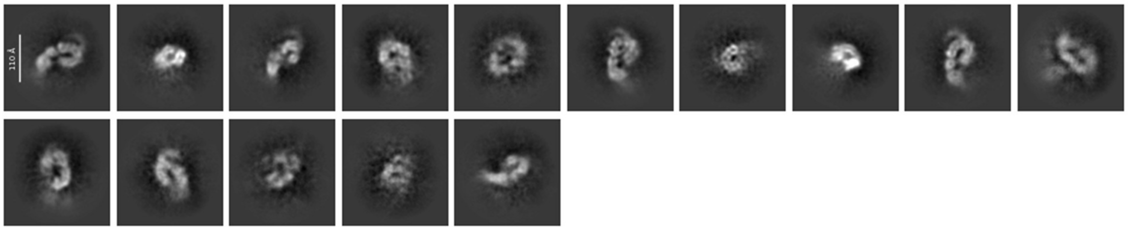

High-resolution 2D class images of lipid-free myoferlin (1-1997)

Generated with the cryosparc-tools python script: https://tools.cryosparc.com/examples/hi-res-2d-classes.html

Fig EV3 B

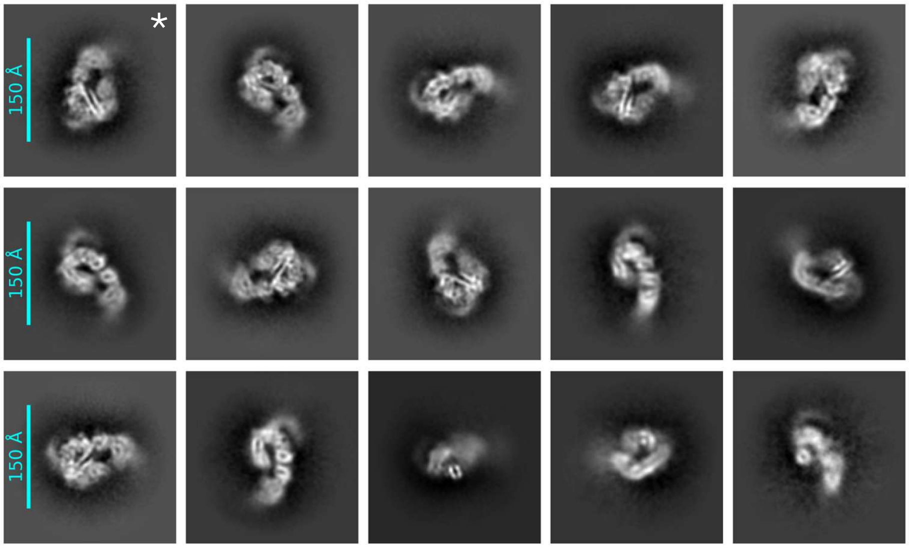

Appendix Fig S4C: Myoferlin (1-1997)-MSP2N2 complex

(nanodisc composition: 25 mol% DOPS, 5 mol% PI $(4,5)P_2$, 5 mol% Cholesterol)

2D classification job (job id: J6749, cryoSPARC v.4.5.1), 40 2D classes, 506,553 particles

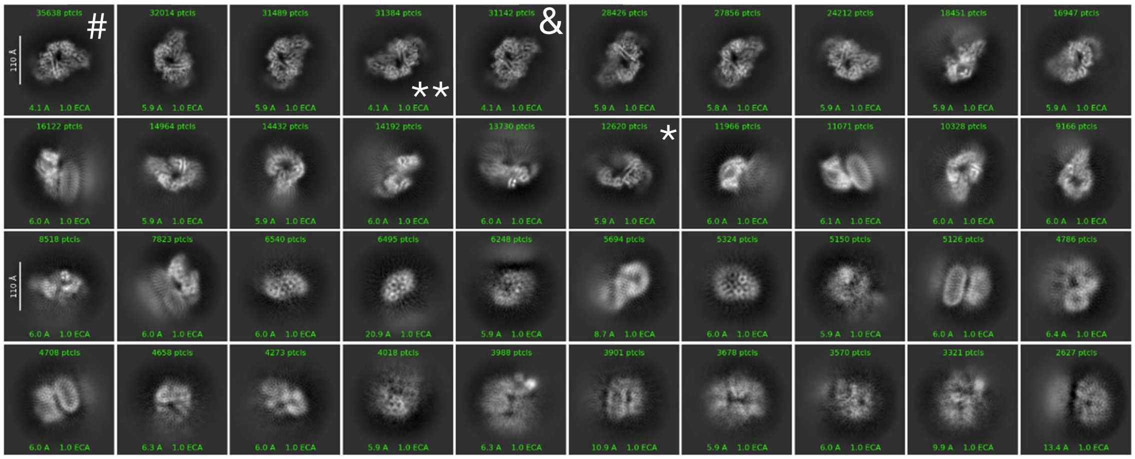

***12,620 particles; **31,384 particles;**
# 35,638 particles; & 31,142 particles

Selected 2D classes: 20

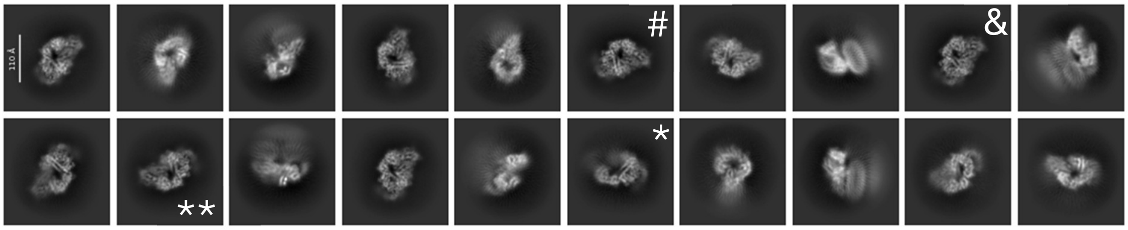

Omitted 2D classes: 20

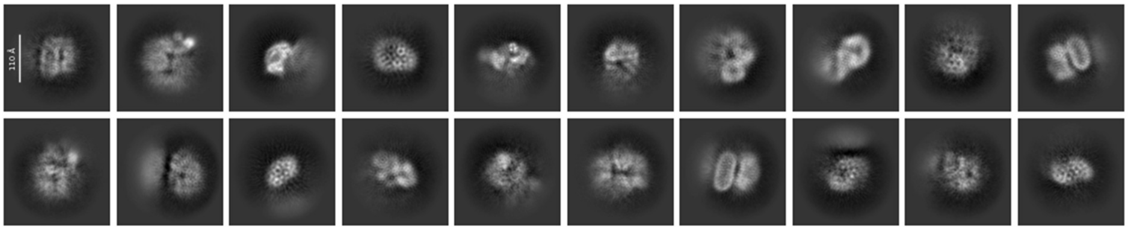

High-resolution 2D class images of myoferlin (1-1997)-MSP2N2 complex

(nanodisc composition: 25 mol% DOPS, 5 mol% PI(4,5)P_2, 5 mol% Cholesterol)

Generated with the cryosparc-tools python script: https://tools.cryosparc.com/examples/hi-res-2d-classes.html

Appendix Fig S4C

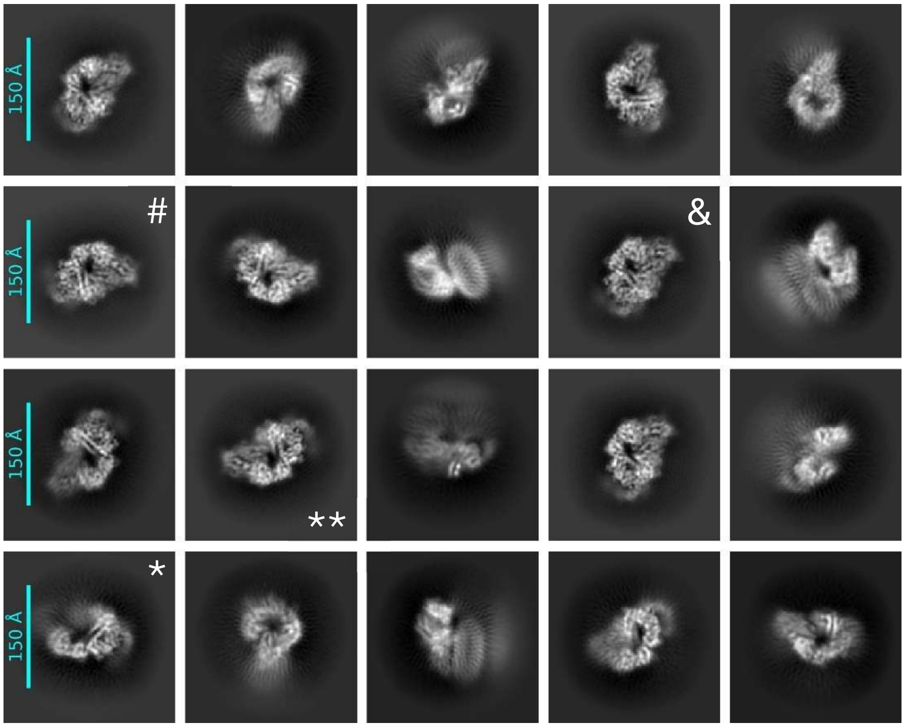

*represents a 2D average of lipid-free myoferlin particles which were observed in the myoferlin (1-1997)-MSP2N2 (25 mol% DOPS, 5 mol% PI(4,5)P_2, 5 mol% Cholesterol nanodisc) dataset, possibly resulting from nanodisc dissociation or weak nanodisc binding.

#, **and &– represent two independently calculated 2D class averages of myoferlin (1-1997)-MSP2N2.

Appendix Fig S2B: Myoferlin (1-1997)-MSP2N2 complex

(nanodisc composition: 25 mol% DOPS, 5 mol% PI $(4,5)P_2$)

2D classification job (job id: J5738, cryoSPARC v.4.5.1), 40 2D classes, 254,949 particles

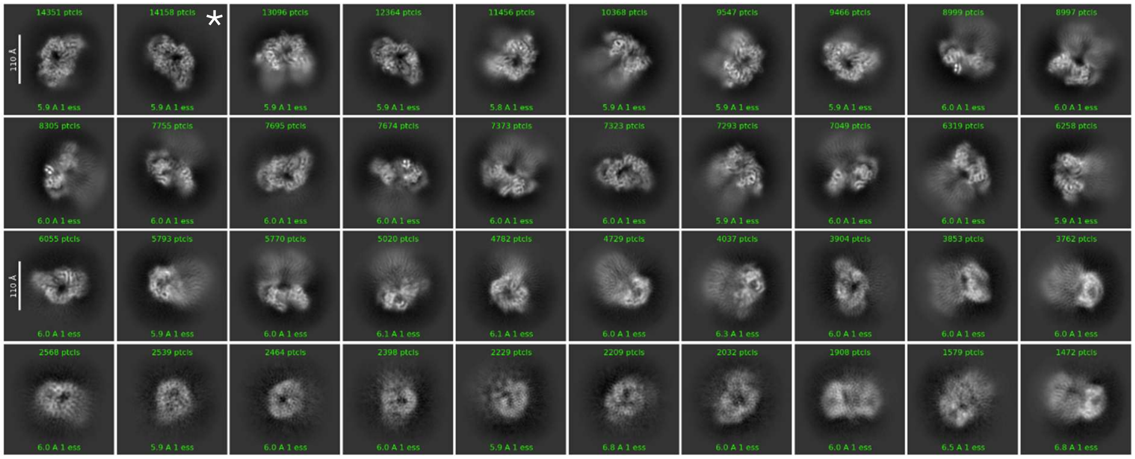

*14,158 particles

Selected 2D classes: 20

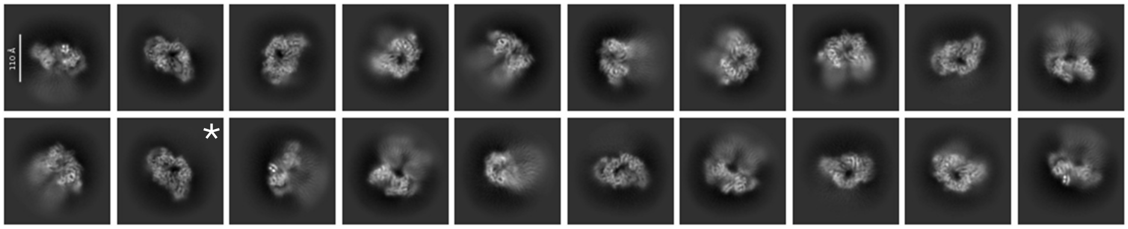

Omitted 2D classes: 20

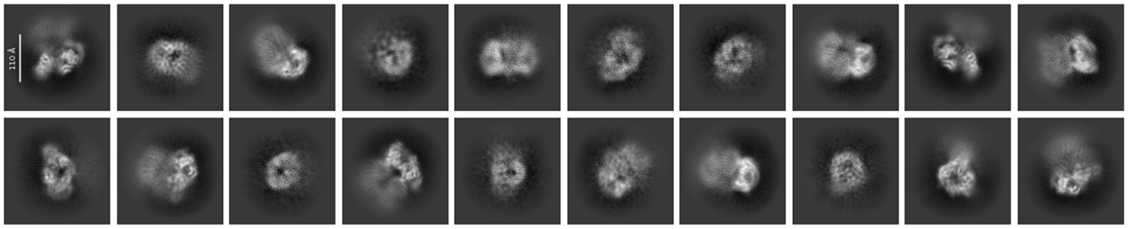

High-resolution 2D class images of myoferlin (1-1997)-MSP2N2 complex

(nanodisc composition: 25 mol% DOPS, 5 mol% PI(4,5)P_2 nanodisc)

Generated with the cryosparc-tools python script: https://tools.cryosparc.com/examples/hi-res-2d-classes.html

Appendix Fig S2B (top panel)

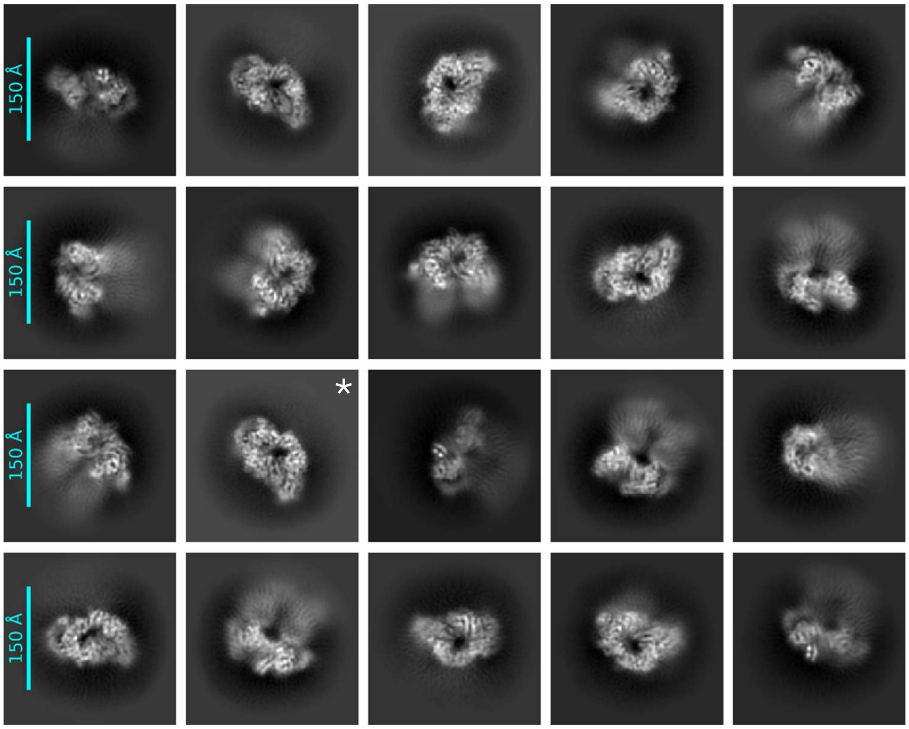

Appendix Fig S2B (bottom panel)

In Appendix Fig 2B (bottom panel) we provide "close-up" views of two 2D class averages (marked with an asterisk, see also below) to allow for better visualization in 2D of the modelled C_2 domains. The original 2D class images used to generate the left and right panels of S2C are shown below, with asterisks marking the 2D classes displayed in the blow-up views.

S2C (left panel, former S2B left bottom panel)

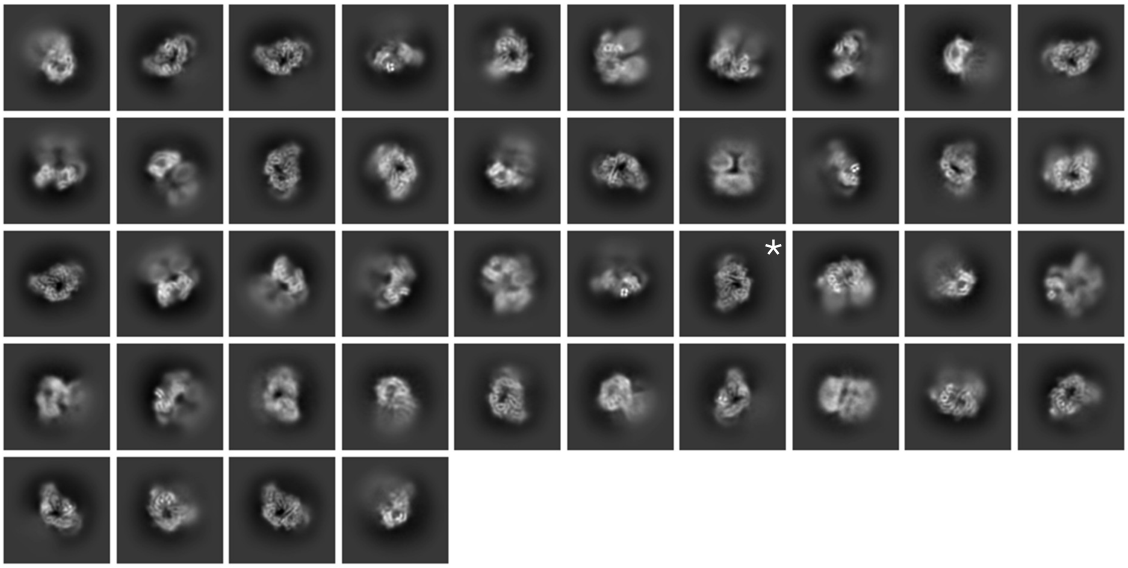

S2C (right panel, former S2B bottom panel)

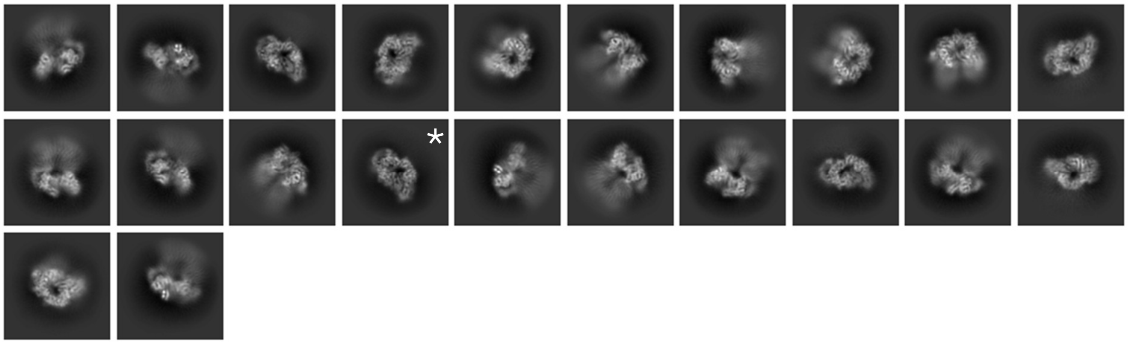

Appendix Figure S2

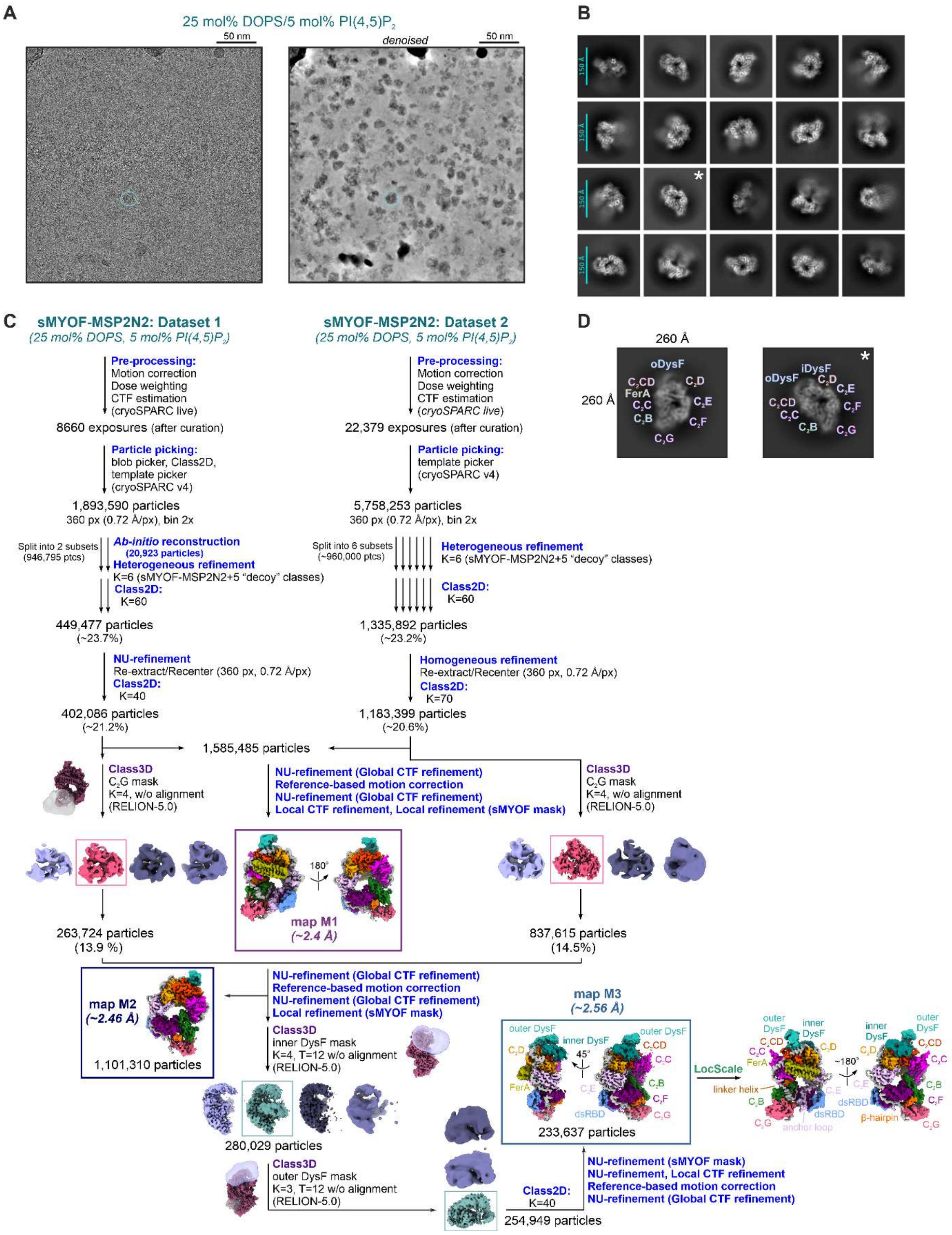

Appendix Figure S2. Computational image analysis of the vitrified myoferlin-MSP2N2 nanodisc complex (25 mol% DOPS and 5 mol% $PI(4,5)P_2$ cryo-EM datasets).

A Cryo-EM micrograph of the vitrified myoferlin (1-1997)-MSP2N2 complex (25 mol% DOPS and 5 mol% $PI(4,5)P_2$ nanodisc). A characteristic, monomeric particle is circled in cyan, in both the raw (left) and denoised (right) exposure.

B Reference-free 2D class averages of the myoferlin (1-1997)-MSP2N2 complex (comprising 25 mol% DOPS and 5 mol% $PI(4,5)P_2$). High-resolution 2D class averages of the sample were generated with the cryosparc-tools package. The 2D average marked with an asterisk is shown as a "close-up" view in **C**.

C Cryo-EM data processing routine of the lipid-bound myoferlin, assembled on a 25 mol% DOPS/5 mol% $PI(4,5)P_2$ MSP2N2 nanodisc (see also Appendix Fig S1B-C). The final maps are boxed and coloured after the modelled domains (*e.g.*, as in Fig 1).

D Close-up views of two characteristic 2D class averages of myoferlin (1-1997)-MSP2N2 complex (25 mol% DOPS, 5 mol% $PI(4,5)P_2$ nanodisc). The visible myoferlin domains are indicated and colour coded.

Appendix Fig S5B: Myoferlin (1-1997)-MSP2N2 complex

(nanodisc composition: 15 mol% DOPS, 5 mol% Cholesterol)

2D classification job (job id: J6284, cryoSPARC v.4.5.1), 30 2D classes, 278,007 particles

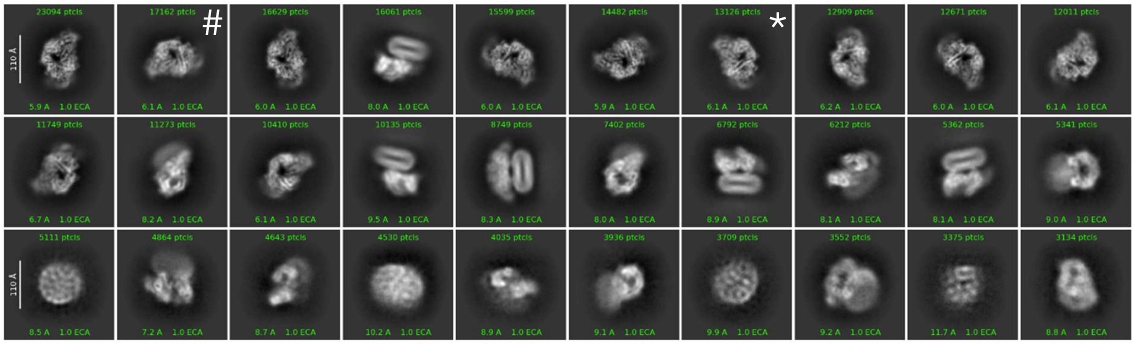

*13,126 particles; #17,162 particles

Selected classes: 20

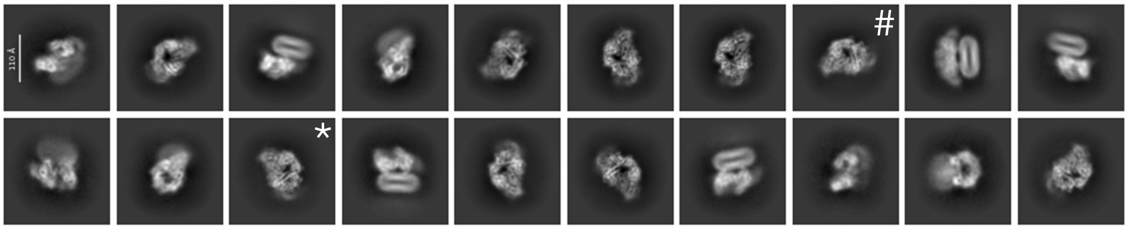

Omitted classes: 10

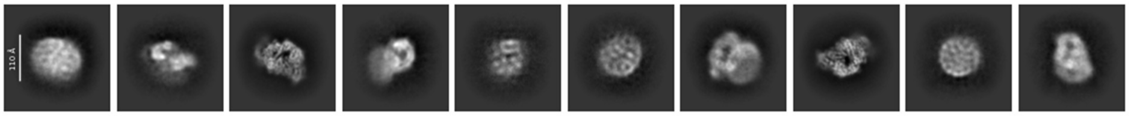

High-resolution 2D class images of myoferlin (1-1997)-MSP2N2 complex

(nanodisc composition: 15 mol% DOPS, 5 mol% Cholesterol)

Generated with the cryosparc-tools python script: https://tools.cryosparc.com/examples/hi-res-2d-classes.html

Appendix Fig 5B

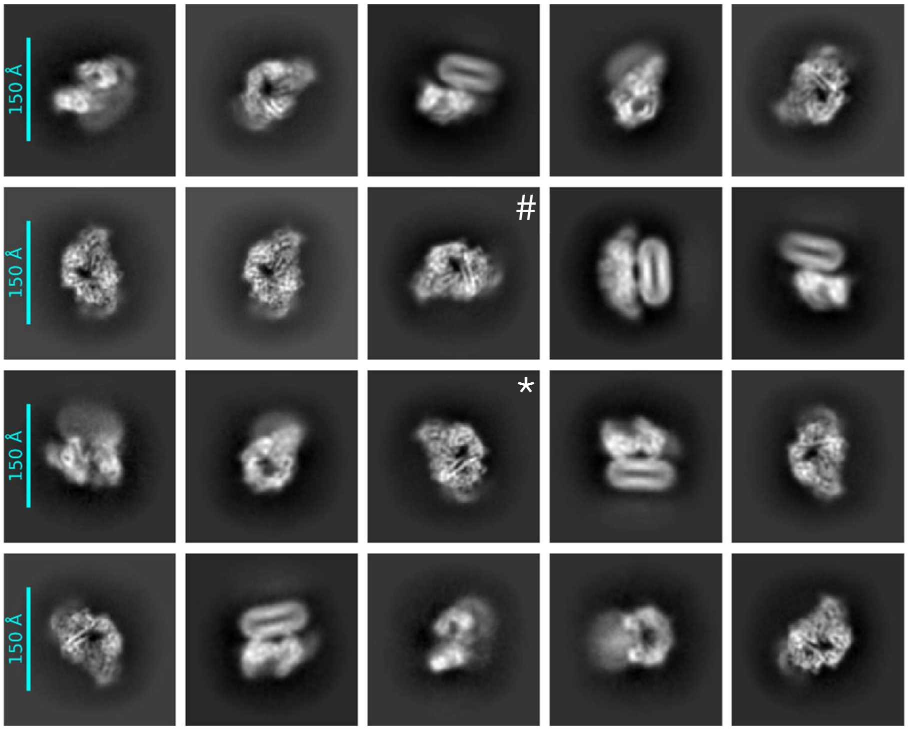

Appendix Fig S5E: Myoferlin (1-1997)-MSP2N2 complex

(nanodisc composition: 15 mol% DOPS, 2 mol% $PI(4,5)P_2$)

2D classification job (job id: J5902, cryoSPARC v.4.5.1), 60 2D classes, 886,710 particles

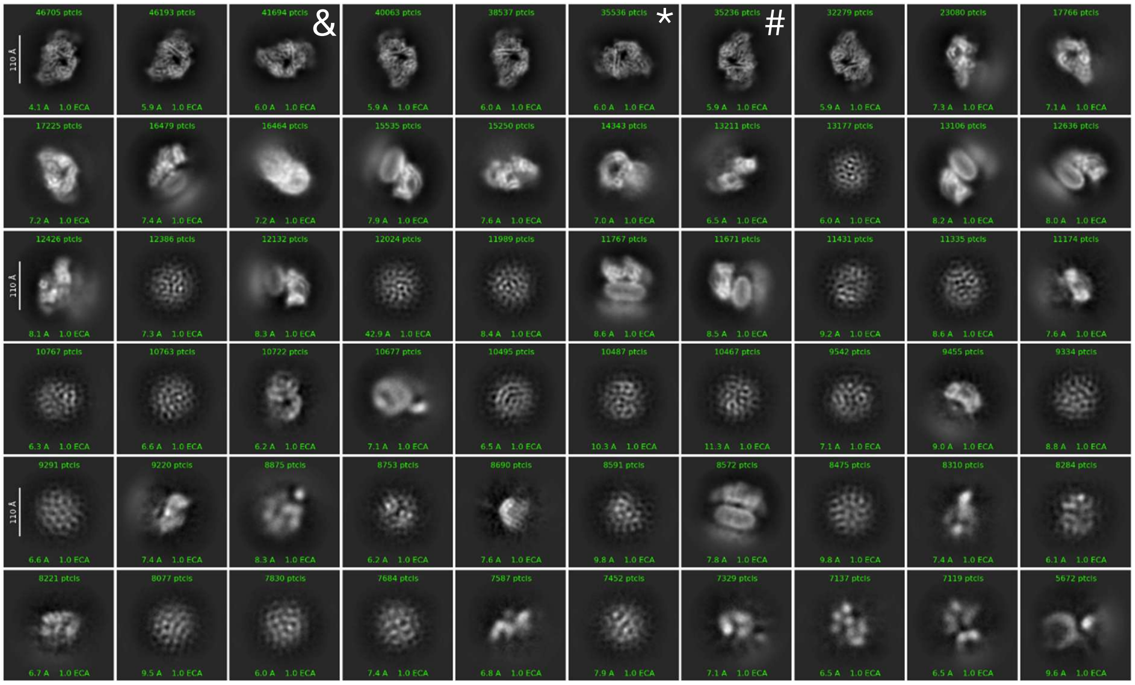

***35,536 particles; & 41,694 particles;# 35,236 particles**

Selected classes: 20

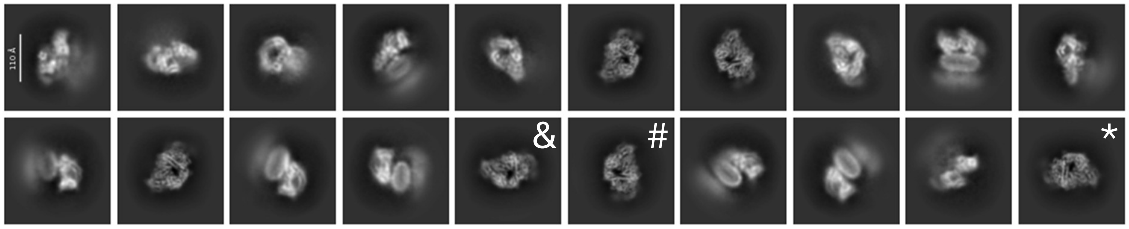

Omitted classes: 40

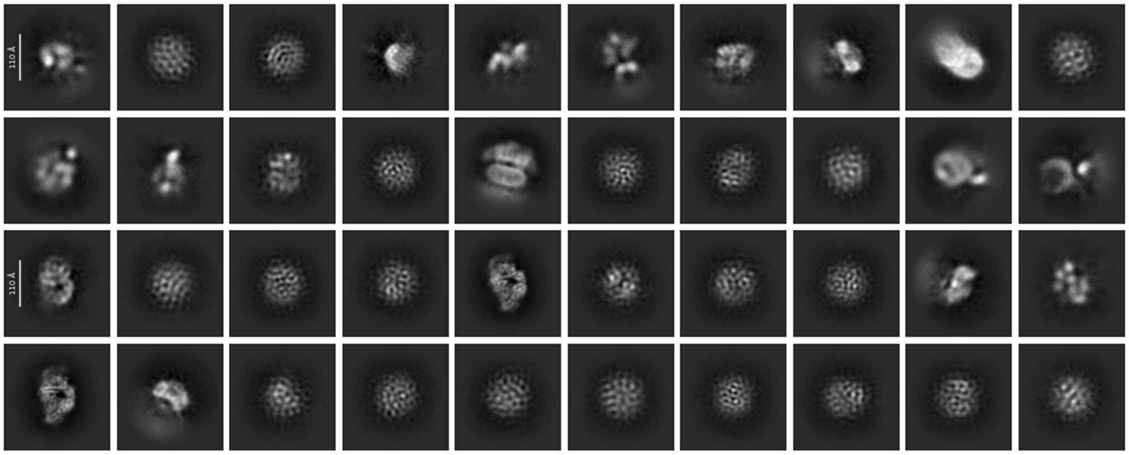

High-resolution 2D class images of myoferlin (1-1997)-MSP2N2 complex

(nanodisc composition: 15 mol% DOPS, 2 mol% PI(4,5)P_2)

Generated with the cryosparc-tools python script: https://tools.cryosparc.com/examples/hi-res-2d-classes.html

Appendix Fig 5E

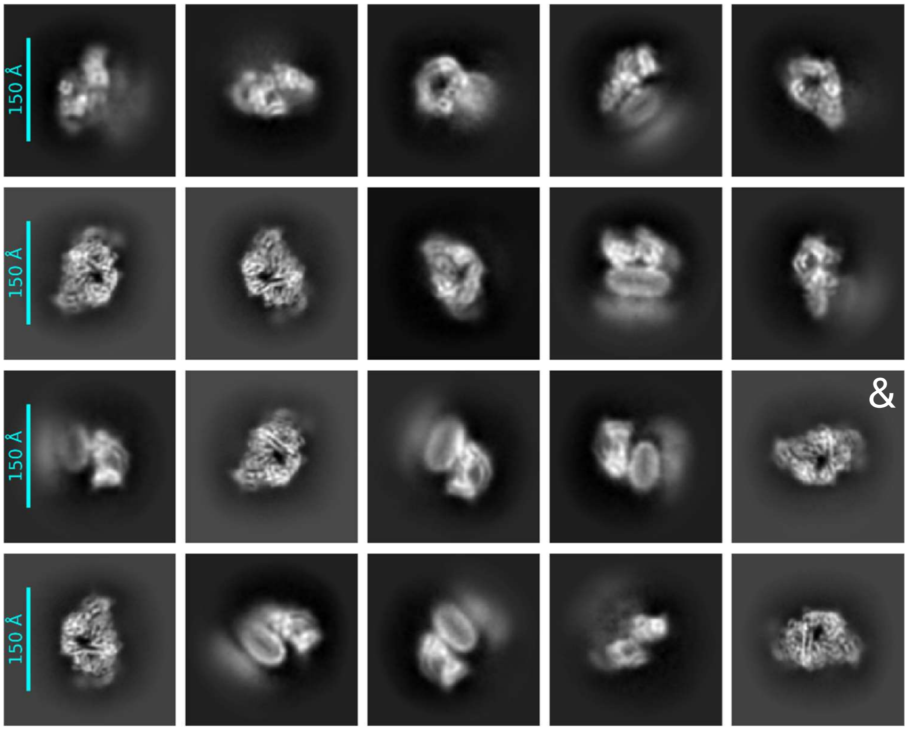

Supplement: Supplementary file 9 — Figure EV3 and Appendix Source Data (2D class averages) [file 44318_2025_463_MOESM9_ESM.pdf]
