# Supplementary material for: Structural insights into lipid membrane binding by human ferlins
Source: EMBO J. 2025 May 28;44(14):3926–58. doi: 10.1038/s44318-025-00463-8 (PMC12264198; doi:10.1038/s44318-025-00463-8)

Figure S1A

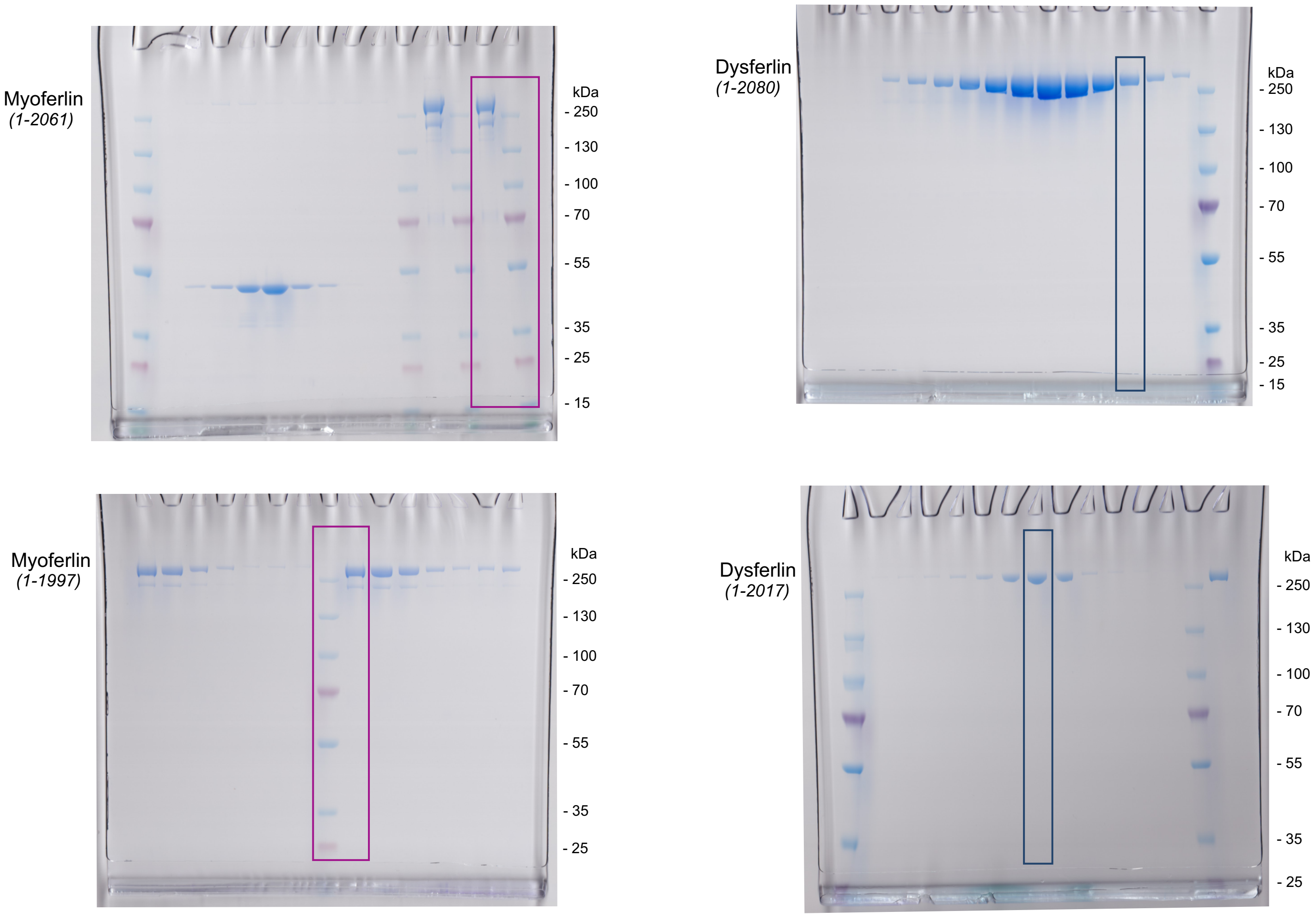

Fig S1C

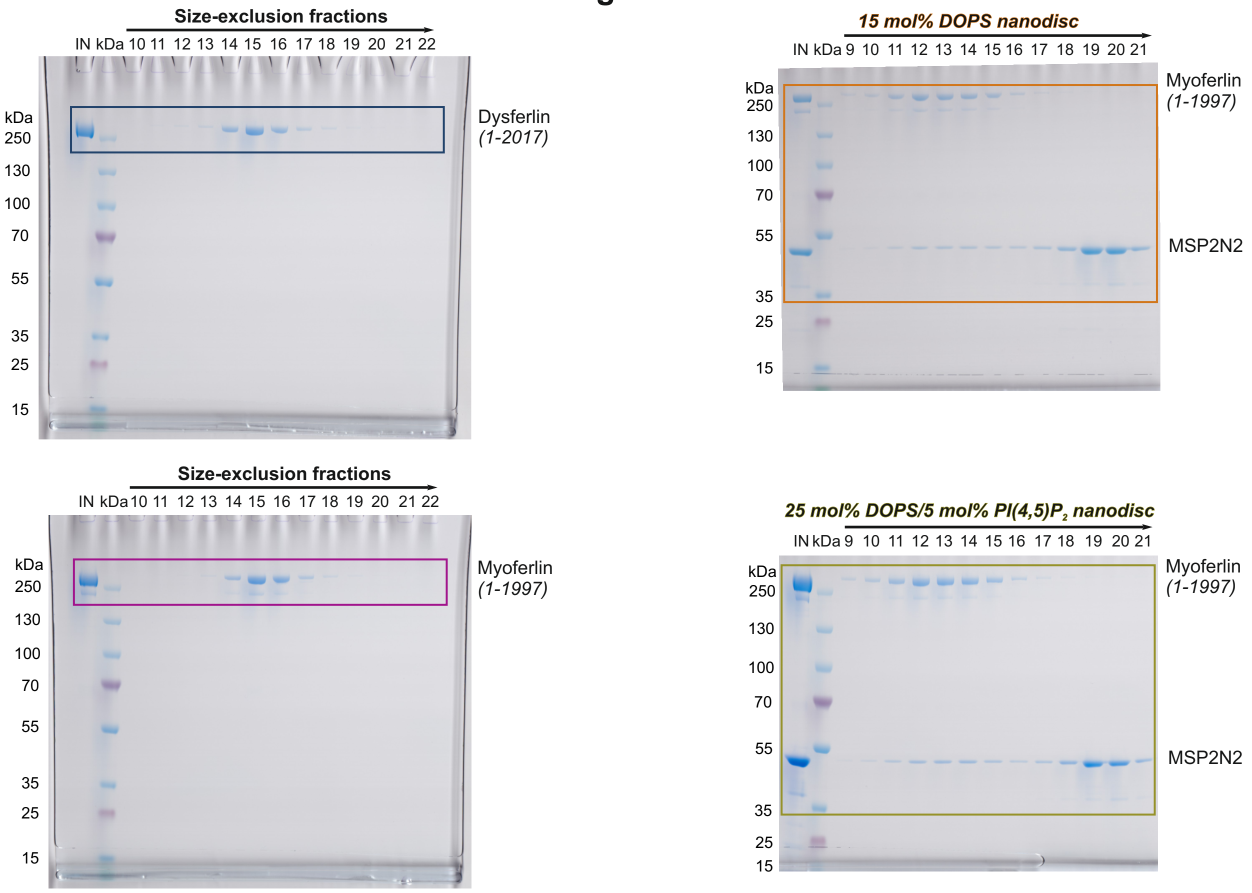

Fig S1D

25 mol% DOPS/1 mol% PI(4,5)P$_2$

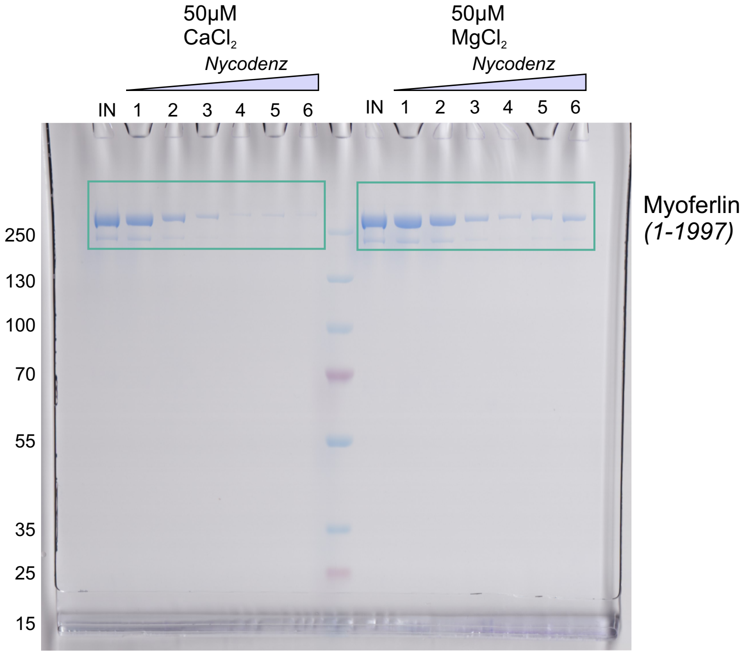

15 mol% DOPS/0.4 mol% PI(4,5)P$_2$

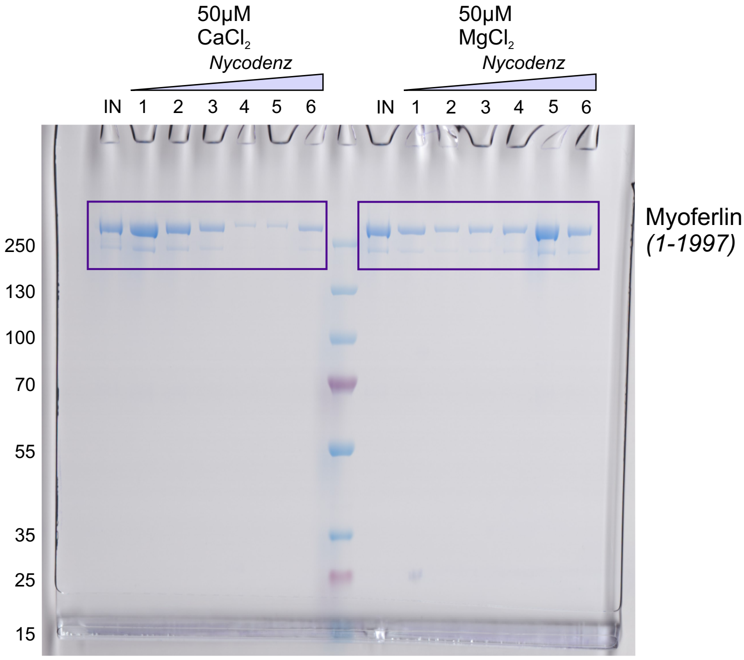

Fig S1D

DOPC/DOPE-only

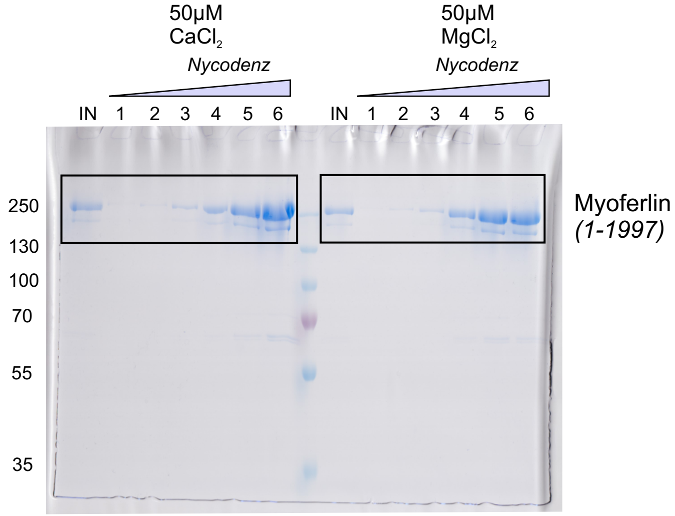

Fig S1H

Binding events (single frame of the recording)

Dysferlin (1-2080)-LMNG: no Ca^{2+}

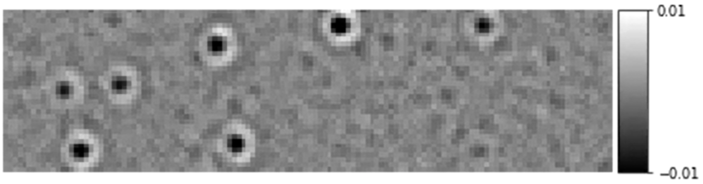

Dysferlin (1-2080)-LMNG: 2 mM Ca^{2+}

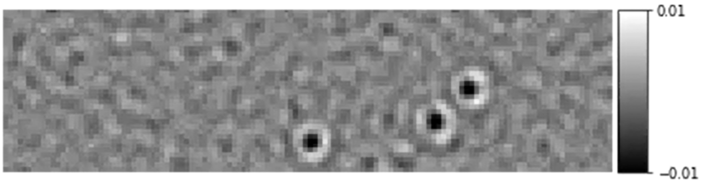

Dysferlin (1-2080)-LMNG: 4 mM Ca^{2+}

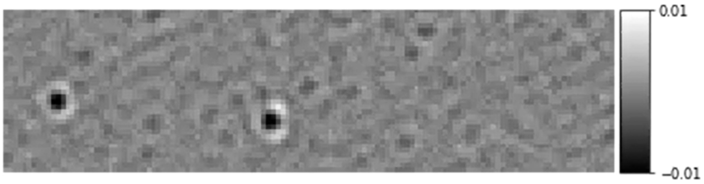

Fig EV4F

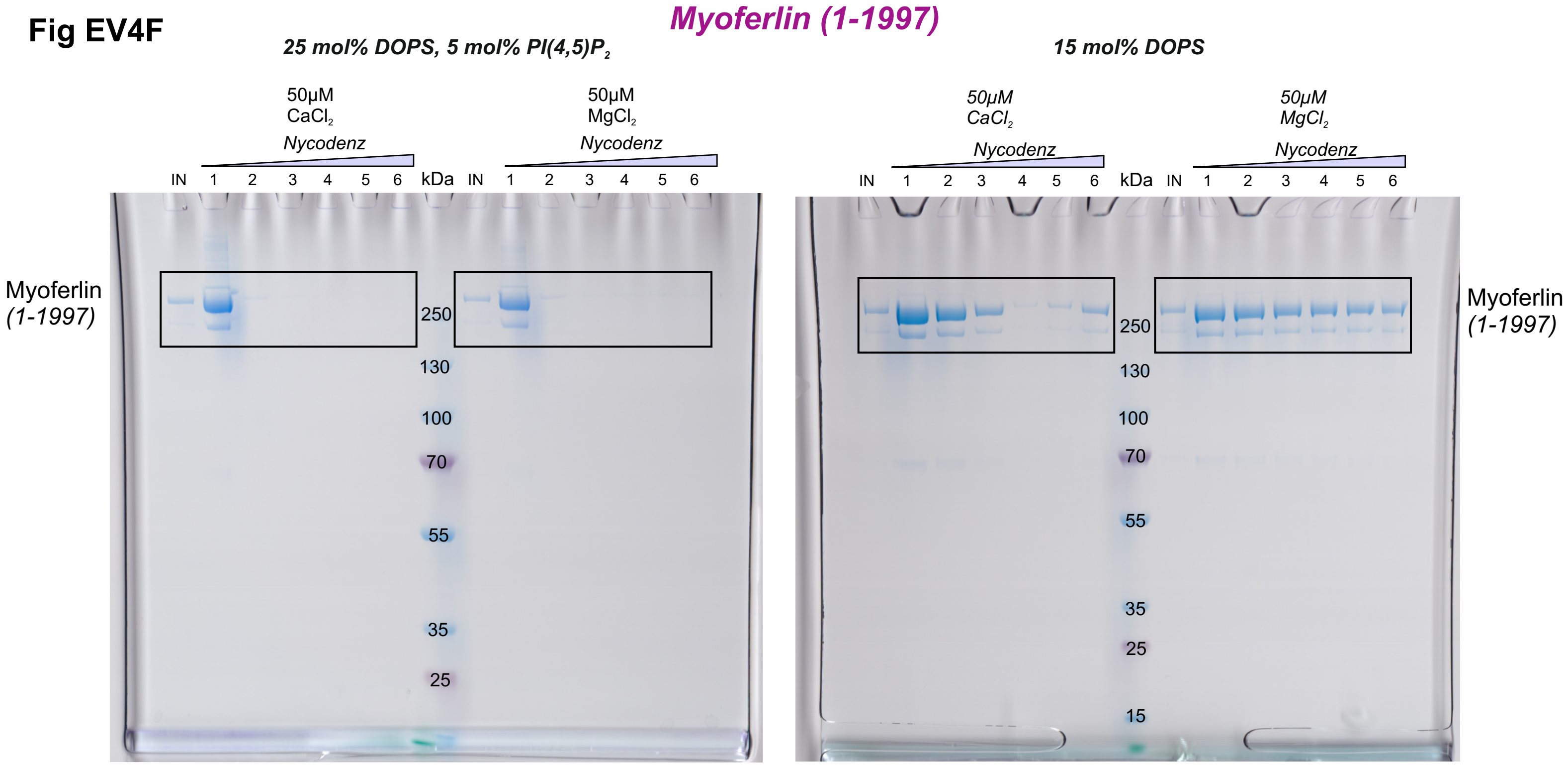

Fig EV4G

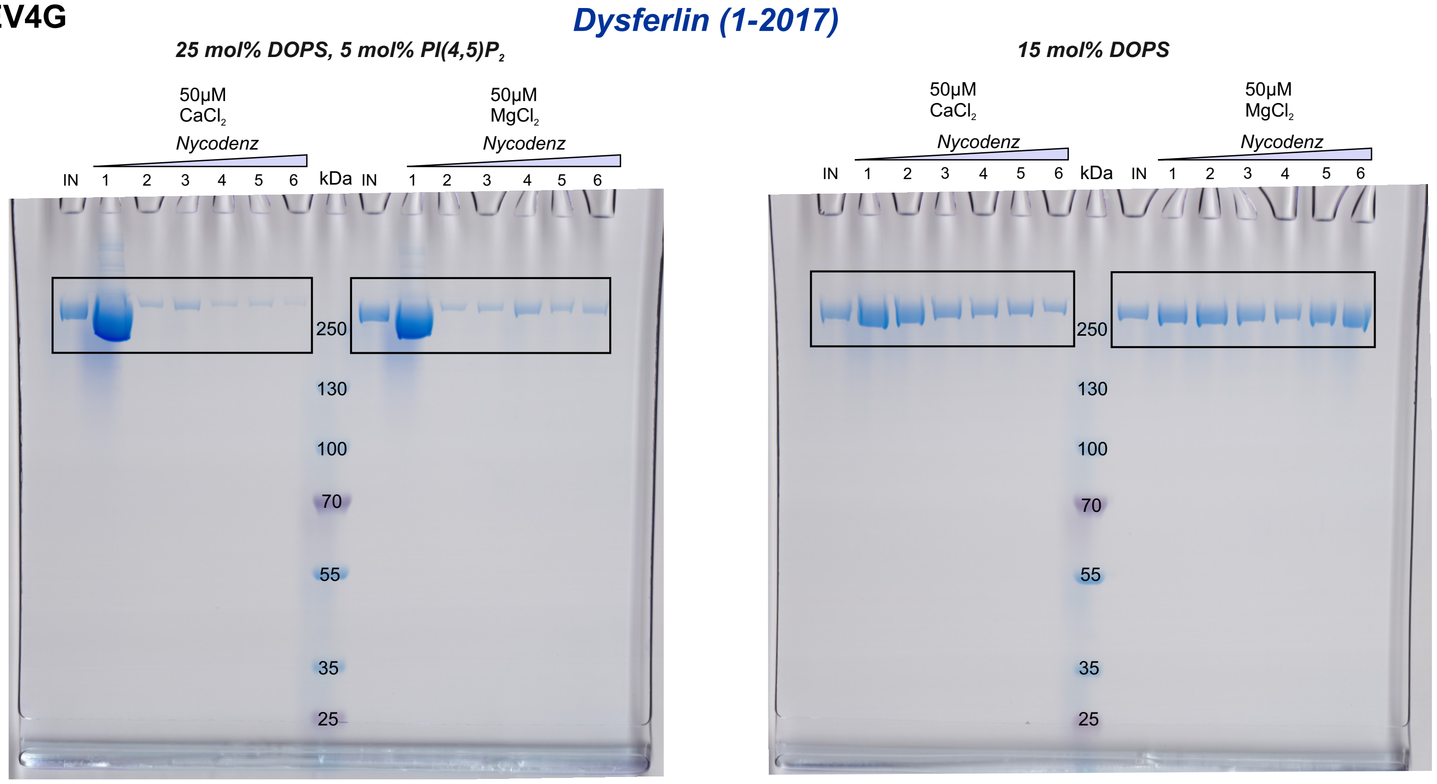

Fig S12F

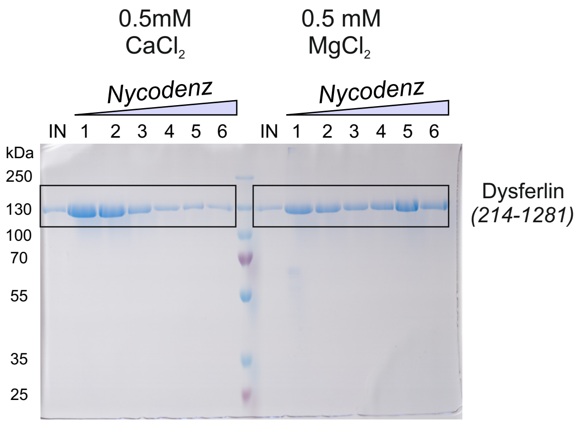

Fig S12F

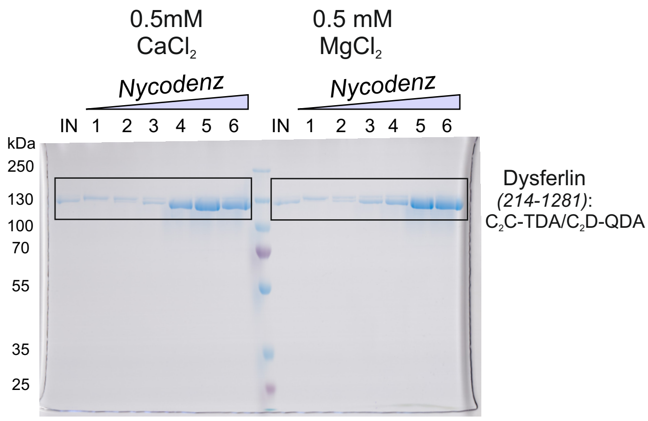

Fig S13D

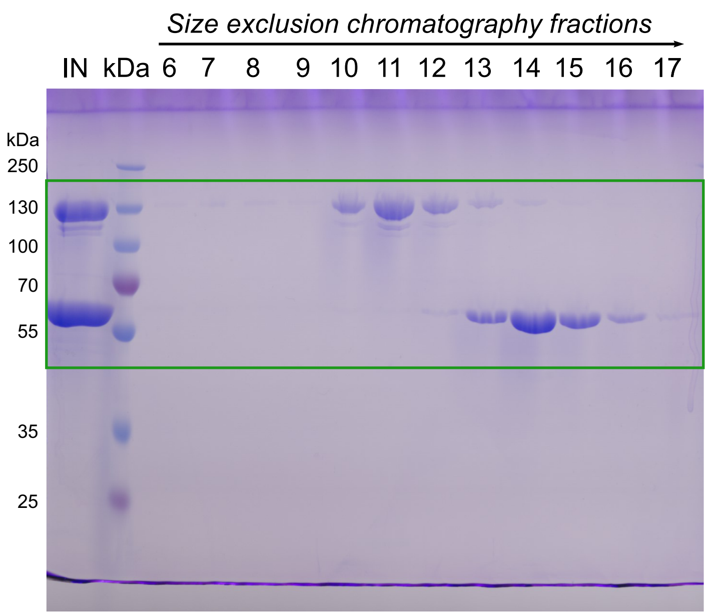

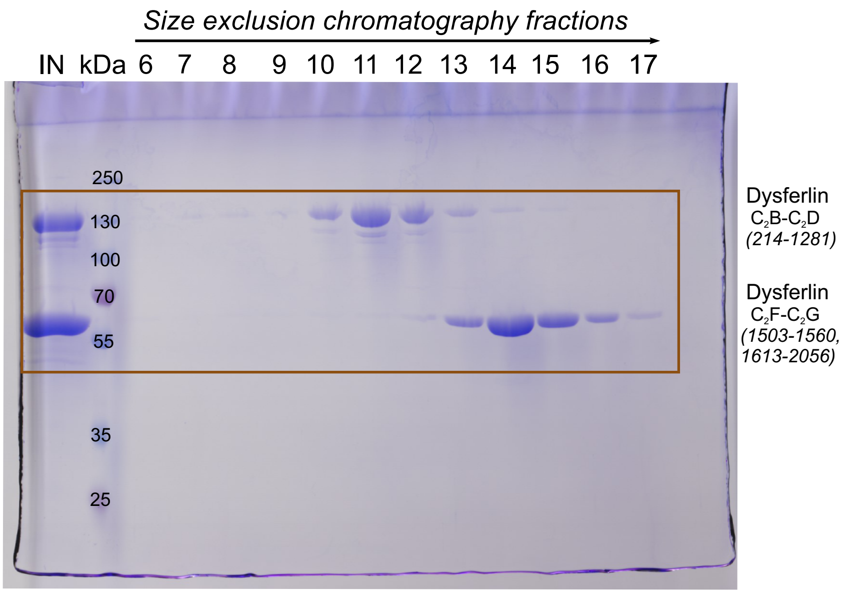

Fig S13D

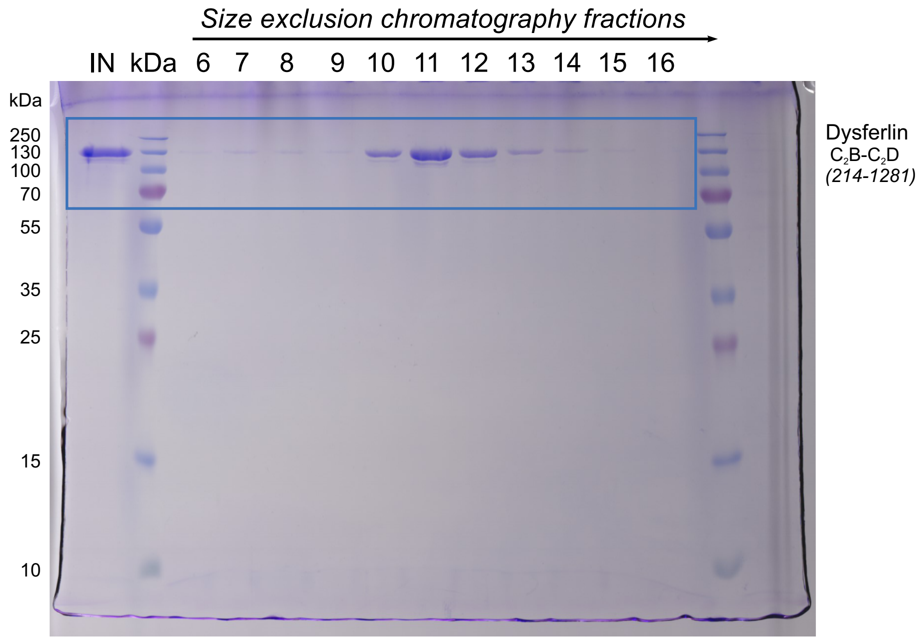

Fig S13D

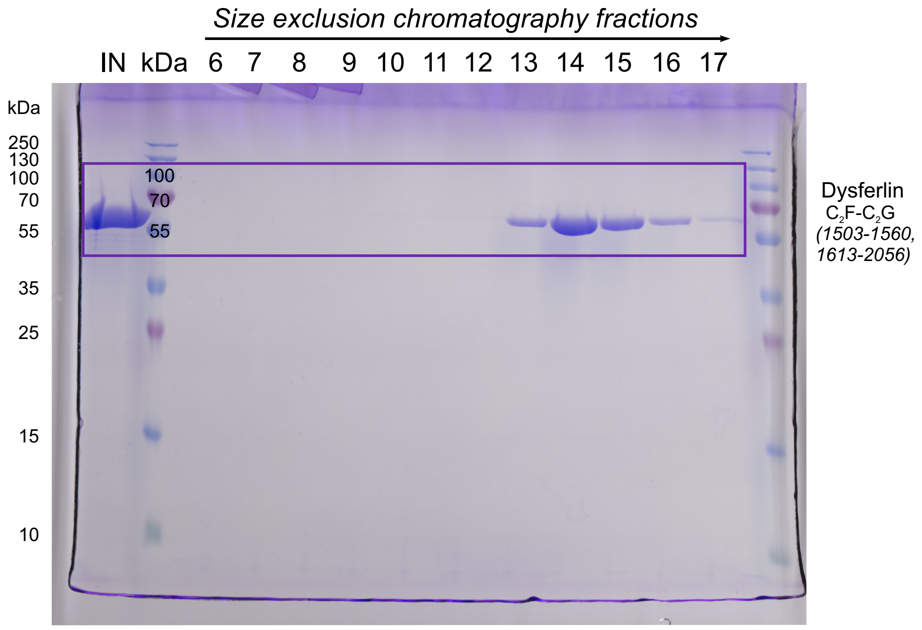

Fig S13A

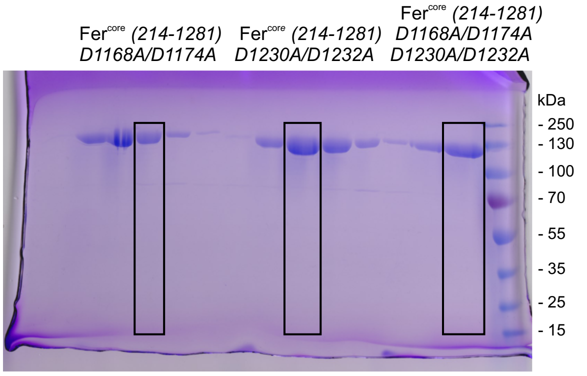

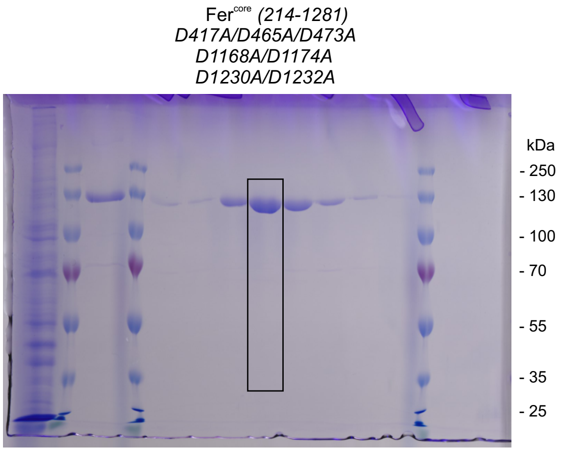

Supplement: Supplementary file 10 — Figure EV4 Source Data [file 44318_2025_463_MOESM10_ESM.pdf]
