# Supplementary material for: Structural insights into lipid membrane binding by human ferlins
Source: EMBO J. 2025 May 28;44(14):3926–58. doi: 10.1038/s44318-025-00463-8 (PMC12264198; doi:10.1038/s44318-025-00463-8)
Supplement: Supplementary file 12 — Expanded View Figures [file 44318_2025_463_MOESM12_ESM.pdf]

Expanded View Figures

Figure EV1. Cryo-EM density snapshots of lipid-bound soluble myoferlin (1–1997).

(**A**) Selected cryo-EM density snapshots of C_2B and its proximal motifs (the linker helix and the FerI motif). Myoferlin's residues are depicted as sticks. (**B**) Cryo-EM density of myoferlin's C_2C domain. The three bound Ca^{2+}-ions and the recruited phosphatidylserine (PS) are indicated. (**C**) Ca^{2+}-binding sites of the C_2C domain of myoferlin. The cryo-EM density is contoured around the Ca^{2+}-binding residues of C_2C. (**D**) Cryo-EM density of the FerA motif of myoferlin. Notably, the four-helix bundle domain inserts between the β4 and β5 strands of the C_2CD domain (red, shown in a cartoon representation). (**E**) Cryo-EM density of the C_2CD domain of myoferlin. The seven β-strands of the domain are indicated. (**F**) Cryo-EM densities of the modelled inner and outer DysF motifs of myoferlin. (**G**) Cryo-EM density and the observed Ca^{2+}-binding sites of the C_2D domain of myoferlin. C_2D's cryo-EM density is contoured around the Ca^{2+}-binding sites (bottom panel). (**H**) Density of the C_2E domain. The domain comprises an extended insertion loop (residues 1395–1498, denoted as the anchor loop), inserted between the β6-β7 strands. The loop establishes contacts with the downstream C_2F domain. (**I**) Cryo-EM density of C_2F and its Ca^{2+}- and phospholipid-binding sites. Three Ca^{2+} ions and a PS headgroup were identified in the cryo-EM density map. (**J**) Cryo-EM density of the C-terminal C_2G domain, derived from the myoferlin (1–1997)-nanodisc complex containing 25 mol% DOPS and 5 mol% $PI(4,5)P_2$. The C_2G domain is shown in two orientations, with its lipid-binding β-hairpin motif coloured light orange. (**K**) Density of the C-terminal C_2G domain, resolved in the myoferlin (1–1997)-nanodisc complex containing 15 mol% DOPS and 2 mol% $PI(4,5)P_2$. Notably, the insertion loop of C_2G (residues 1906–1944) is ordered in this myoferlin complex. (**L**) Ca^{2+}-binding sites of C_2G, observed in the lipid-bound myoferlin structure (15 mol% DOPS and 2 mol% $PI(4,5)P_2$ nanodisc). Due to the lower local resolution, Ca^{2+} modelling was initiated by AlphaFold3 predictions (Abramson et al, 2024), and the optimal sites were refined against the cryo-EM map of the complex (Appendix Fig. S5F). Our modelling (right panel) suggests that two Ca^{2+} ions are coordinated by the L1 and L3 loops of C_2G (Corbalan-Garcia and Gomez-Fernandez, 2014; Rizo and Sudhof, 1998). (**M**) Cryo-EM densities of the Ca^{2+}-bound phosphatidylserine (PS), resolved in the myoferlin-nanodisc complexes. The cryo-EM density is contoured around the PS headgroups, and the ligands are depicted as sticks.

 © The Author(s)

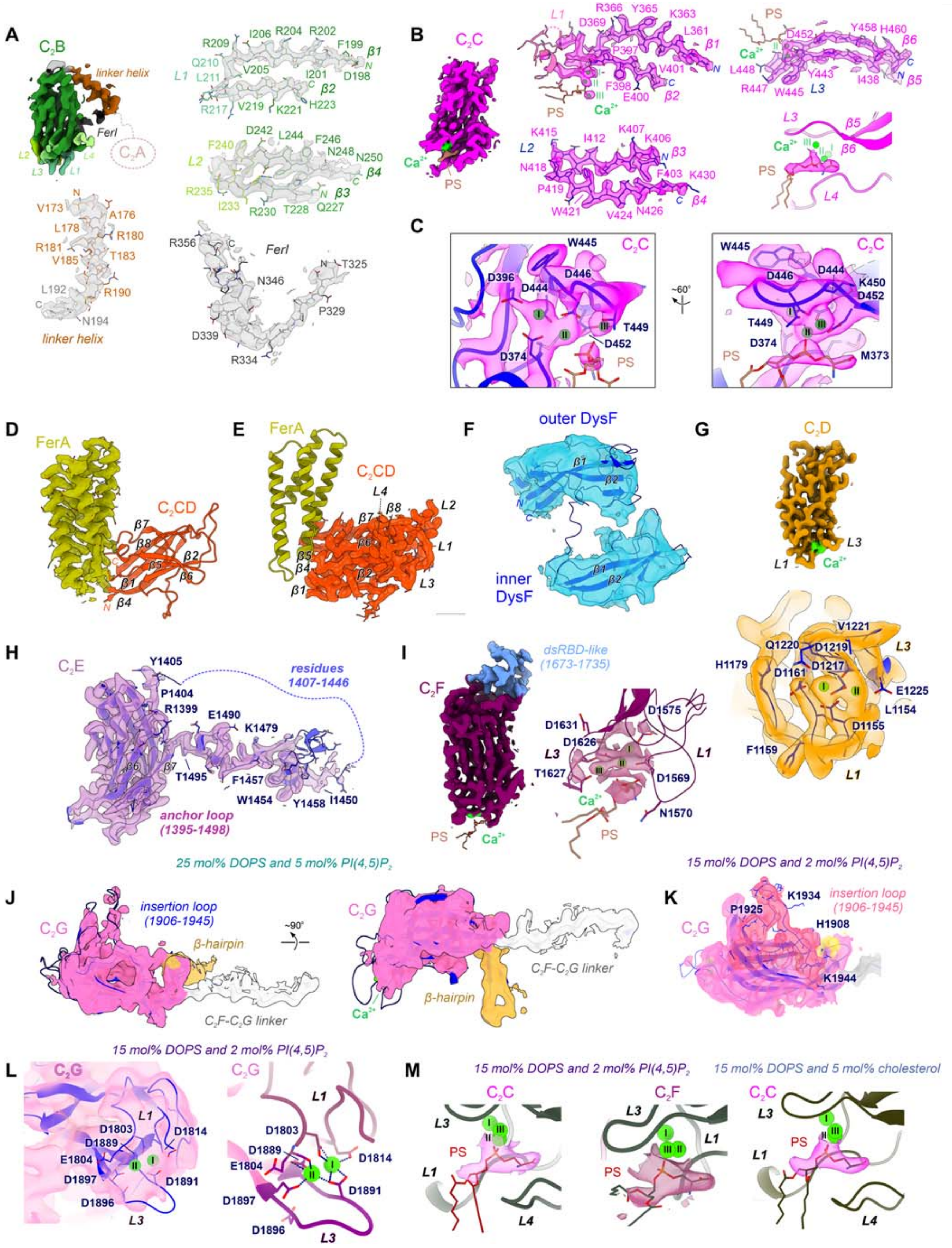
A
C2B
linker helix
FerI
C2A
L1 L2 L3 L4
R209 I206 R204 R202 F199 β1
Q210 V205 I201 D198
L211 β2
R217 V219 K221 H223
D242 L244 F246
F240 N248 N250
L2 β4
R235 β3
I233 R230 T228 Q227
V173 A176 L178 R180 R181 T183 V185 L192 R190 N194
linker helix
R356 FerI T325 N346 P329 D339 R334
B
C2C
Ca2+ PS
L1 R366 Y365 K363 D369 L361 β1 P397 V401 F398 E400 β2
PS Ca2+
K415 K407 I412 K406 L2 β3 N418 F403 K430 P419 β4 W421 V424 N426
PS D452 Y458 H460 β6 Ca2+ L448 Y443 I438 β5 R447 W445 L3
L3 β5 β6 Ca2+ PS L4
C
W445 C2C D396 D446 D444 T449 D452 D374 PS
~60°
W445 C2C D446 D444 K450 D452 T449 D374 M373 PS
D
FerA C2CD β7 β8 β2 β5 β6 β1 β4
E
FerA C2CD L4 β8 L2 β7 β6 L1 β5 β4 β2 β1 L3
F
outer DysF β1 β2 inner DysF β1 β2
G
C2D L3 L1 Ca2+
V1221 Q1220 D1219 L3 H1179 D1161 D1217 E1225 L1154 D1155 F1159 L1
H
C2E Y1405 residues 1407-1446 P1404 R1399 E1490 K1479 β6 β7 T1495 F1457 W1454 Y1458 I1450 anchor loop (1395-1498)
I
dsRBD-like (1673-1735)
C2F
D1575 D1631 D1626 L3 L1 T1627 D1569 Ca2+ N1570 PS Ca2+ PS
25 mol% DOPS and 5 mol% PI(4,5)P2
J
C2G insertion loop (1906-1945) β-hairpin ~90° C2F-C2G linker
C2G C2F-C2G linker β-hairpin Ca2+
15 mol% DOPS and 2 mol% PI(4,5)P2
K
C2G K1934 insertion loop (1906-1945) P1925 H1908 K1944
15 mol% DOPS and 2 mol% PI(4,5)P2
L
C2G L1 D1803 D1814 D1889 E1804 D1897 D1891 D1896 L3
C2G L1 D1803 D1889 E1804 D1814 D1897 D1891 D1896 L3
M
15 mol% DOPS and 2 mol% PI(4,5)P2
15 mol% DOPS and 5 mol% cholesterol
C2C L3 PS L1 L4
C2F L3 L1 PS
C2C L3 PS L1 L4

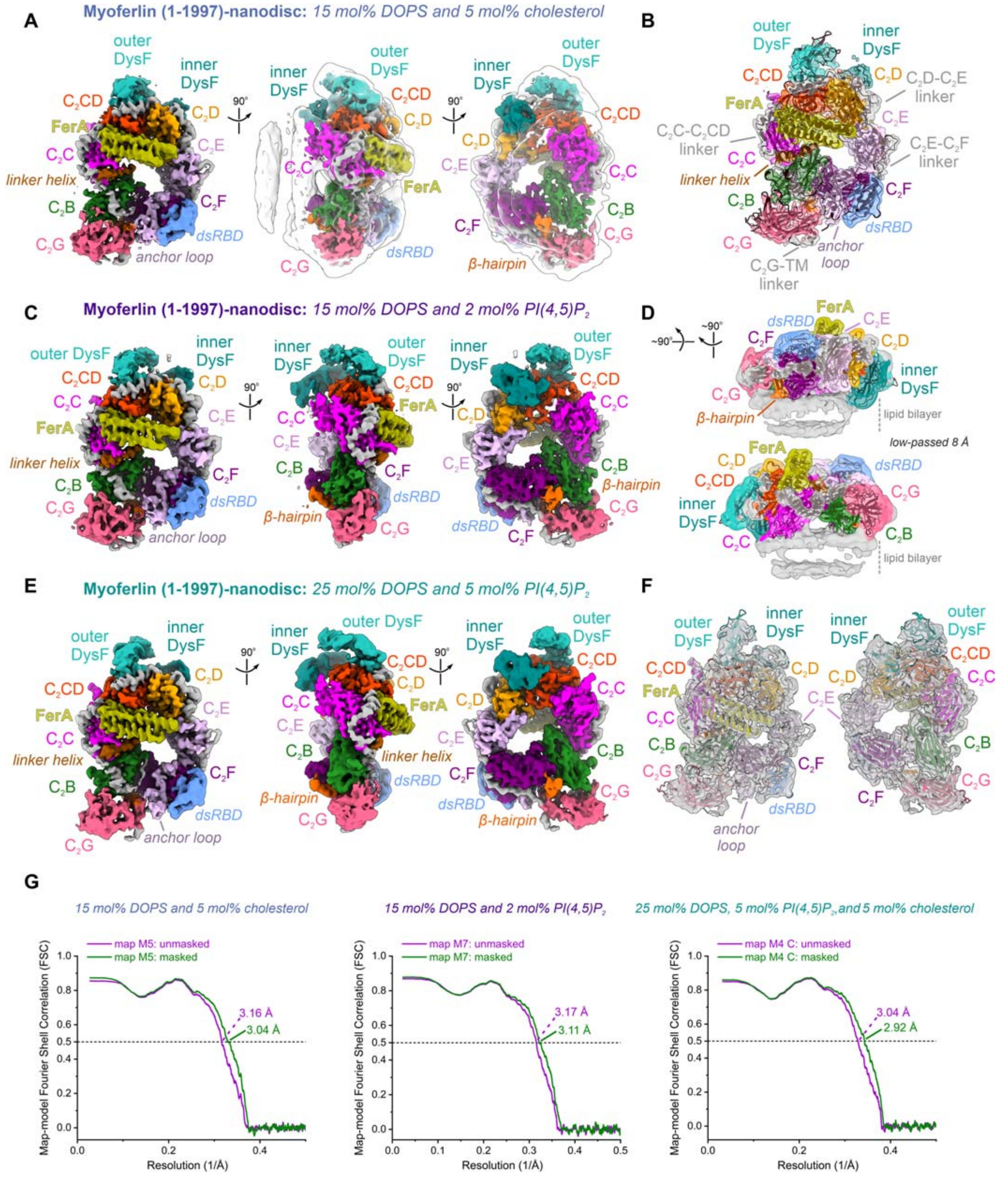
A
Myoferlin (1-1997)-nanodisc: 15 mol% DOPS and 5 mol% cholesterol
B
C
Myoferlin (1-1997)-nanodisc: 15 mol% DOPS and 2 mol% PI(4,5)P2
D
E
Myoferlin (1-1997)-nanodisc: 25 mol% DOPS and 5 mol% PI(4,5)P2
F
outer DysF
inner DysF
C2CD
C2D
FerA
C2C
C2E
linker helix
C2B
C2F
dsRBD
C2G
anchor loop
β-hairpin
C2D-C2E linker
C2C-C2CD linker
C2E-C2F linker
C2G-TM linker
lipid bilayer
low-passed 8 Å
G
15 mol% DOPS and 5 mol% cholesterol
15 mol% DOPS and 2 mol% PI(4,5)P2
25 mol% DOPS, 5 mol% PI(4,5)P2, and 5 mol% cholesterol
map M5: unmasked
map M5: masked
3.16 Å
3.04 Å
map M7: unmasked
map M7: masked
3.17 Å
3.11 Å
map M4 C: unmasked
map M4 C: masked
3.04 Å
2.92 Å
Map-model Fourier Shell Correlation (FSC)
Resolution (1/Å)

 © The Author(s)

Figure EV2. Structural comparison between the different lipid-bound myoferlin complexes.

(**A**) Overall cryo-EM map (map M6, ~3.3 Å) of soluble myoferlin (1–1997) bound to an MSP2N2 nanodisc containing 15 mol% DOPS and 5 mol% cholesterol (Appendix Fig. S5C). The map is shown in three different orientations. A lowpass filtered map (transparent surface) is superimposed to better visualize the nanodisc density. (**B**) Cryo-EM map of the myoferlin (1–1997)-nanodisc complex (15 mol% DOPS and 5 mol% cholesterol nanodisc). The final model is fitted inside the map (Appendix Fig. S5C). Several C_2 domain linker regions, the linker helix, and the anchor loop of C_2E are well resolved in this myoferlin complex. (**C**) Overall cryo-EM map (map M8, ~3.43 Å) of the soluble myoferlin (1–1997) bound to a nanodisc containing 15 mol% DOPS and 2 mol% PI(4,5)P_2 (Appendix Fig. S5F). The map is shown in three different orientations, as in (**A**). (**D**) Side views of the myoferlin (1–1997)-nanodisc complex (map M8, 15 mol% DOPS and 2 mol% PI(4,5)P_2), together with the fitted model (Appendix Fig. S5F). The map was lowpass filtered to 8 Å, and the nanodisc density is indicated. (**E**) Cryo-EM map of the myoferlin (1-1997)-nanodisc complex assembled onto a 25 mol% DOPS and 5 mol% PI(4,5)P_2 MSP2N2 nanodisc (~2.56 Å, map M3, Appendix S2C). (**F**) Overall map of the nanodisc-bound myoferlin (1-1997) complex (map M3, 25 mol% DOPS and 5 mol% PI(4,5)P_2) with the fitted final model. Except for the flexible N-terminal C_2A, all myoferlin domains (C_2B-C_2G) and accessory motifs (FerA, the linker helix, the anchor loop) were accurately built, resulting in the near-complete structure of ferlin's cytosolic region (Appendix Figs. S2C and S3). (**G**) Map versus model Fourier Shell Correlation (FSC) plots for the myoferlin (1–1997)-nanodisc complexes (Appendix Figs. S4A and S5C,F).

© The Author(s)

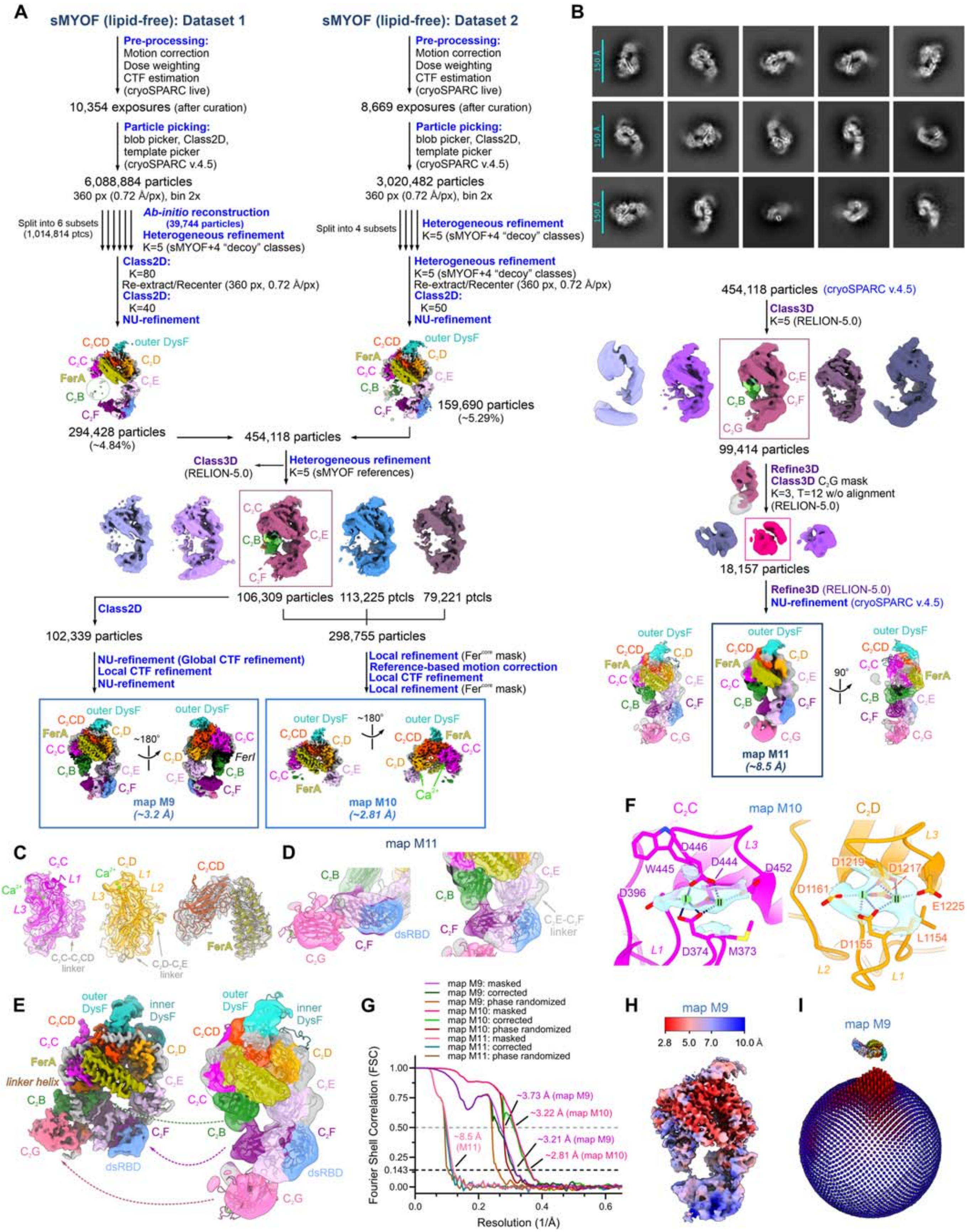
A
sMYOF (lipid-free): Dataset 1
Pre-processing:
Motion correction
Dose weighting
CTF estimation
(cryoSPARC live)
10,354 exposures (after curation)
Particle picking:
blob picker, Class2D,
template picker
(cryoSPARC v.4.5)
6,088,884 particles
360 px (0.72 Å/px), bin 2x
Split into 6 subsets (1,014,814 ptcs)
Ab-initio reconstruction (39,744 particles)
Heterogeneous refinement
K=5 (sMYOF+4 "decoy" classes)
Class2D:
K=80
Re-extract/Recenter (360 px, 0.72 Å/px)
Class2D:
K=40
NU-refinement
294,428 particles (~4.84%)
sMYOF (lipid-free): Dataset 2
8,669 exposures (after curation)
3,020,482 particles
Split into 4 subsets
Heterogeneous refinement
K=5 (sMYOF+4 "decoy" classes)
Class2D:
K=50
159,690 particles (~5.29%)
454,118 particles
Heterogeneous refinement
K=5 (sMYOF references)
Class3D (RELION-5.0)
106,309 particles 113,225 ptcls 79,221 ptcls
Class2D
102,339 particles
298,755 particles
NU-refinement (Global CTF refinement)
Local CTF refinement
NU-refinement
Local refinement (Fer^core mask)
Reference-based motion correction
Local CTF refinement
Local refinement (Fer^core mask)
map M9 (~3.2 Å)
map M10 (~2.81 Å)
B
454,118 particles (cryoSPARC v.4.5)
Class3D
K=5 (RELION-5.0)
99,414 particles
Refine3D
Class3D C2G mask
K=3, T=12 w/o alignment
(RELION-5.0)
18,157 particles
Refine3D (RELION-5.0)
NU-refinement (cryoSPARC v.4.5)
map M11 (~8.5 Å)
C
D
map M11
E
F
map M10
G
map M9: masked
map M9: corrected
map M9: phase randomized
map M10: masked
map M10: corrected
map M10: phase randomized
map M11: masked
map M11: corrected
map M11: phase randomized
Fourier Shell Correlation (FSC)
Resolution (1/Å)
~3.73 Å (map M9)
~3.22 Å (map M10)
~3.21 Å (map M9)
~2.81 Å (map M10)
~8.5 Å (M11)
H
map M9
2.8 5.0 7.0 10.0 Å
I
map M9

Figure EV3. Cryo-EM image analysis of the vitrified lipid-free soluble myoferlin (1–1997).

(**A**) Cryo-EM image processing schematic for the Ca^{2+}-bound, lipid-free myoferlin (1–1997). The resolution of the final maps (M9, M10, and M11 maps) was estimated according to the gold-standard Fourier Shell Correlation (FSC) criterion of 0.143. The structural domains of myoferlin are colour-coded as in Fig. 1. (**B**) Reference-free 2D class averages of lipid-free myoferlin (1–1997). Note the similarities between the dysferlin (1–2017) (Appendix Fig. S6C) and myoferlin (1–1997) 2D class averages in their lipid-free states. (**C**) Cryo-EM density of myoferlin's C_2C, C_2D, and C_2CD-FerA domains. The myoferlin model is fitted inside, and the Fer^{core} map of myoferlin (map M10) is shown as a transparent surface. (**D**) Cryo-EM density of the C_2F (map M9) and C_2G (map M11) domains as modelled in the lipid-free myoferlin (1–1997) structure. The tertiary interfaces between the C_2F-C_2B and C_2F-C_2G are also observed in the lipid-free dysferlin (1–2017) structure (Appendix Figs. S6A, S7G–J, and S8H,I). (**E**) Side-by-side comparison between the membrane-bound (map M3) and lipid-free (map M11) myoferlin structures. The domains undergoing significant displacement upon nanodisc binding (C_2B, C_2F, and C_2G) are indicated with dashed arrows. (**F**) Ca^{2+}-binding sites observed in the lipid-free myoferlin structure. The cryo-EM density is coloured cyan and shown as a transparent surface. The two modelled Ca^{2+} ions, bound to C_2C and C_2D, are coloured green. (**G**) Global resolution estimates for the lipid-free myoferlin (1–1997) cryo-EM maps using Fourier Shell Correction (FSC) between half-maps. The resolution estimates at the FSC = 0.143 and FSC = 0.5 thresholds are indicated. (**H**) Local resolution of the overall map of the lipid-free soluble myoferlin (map M9). The map regions coloured in red indicate higher resolution. (**I**) Angular distribution of the myoferlin particles contributing to the overall map (map M9). The final cryo-EM map is shown above the 3D angular distribution representation. Relative cylinder height and the red colour indicate a higher number of particle images. Source data are available online for this figure.

© The Author(s)

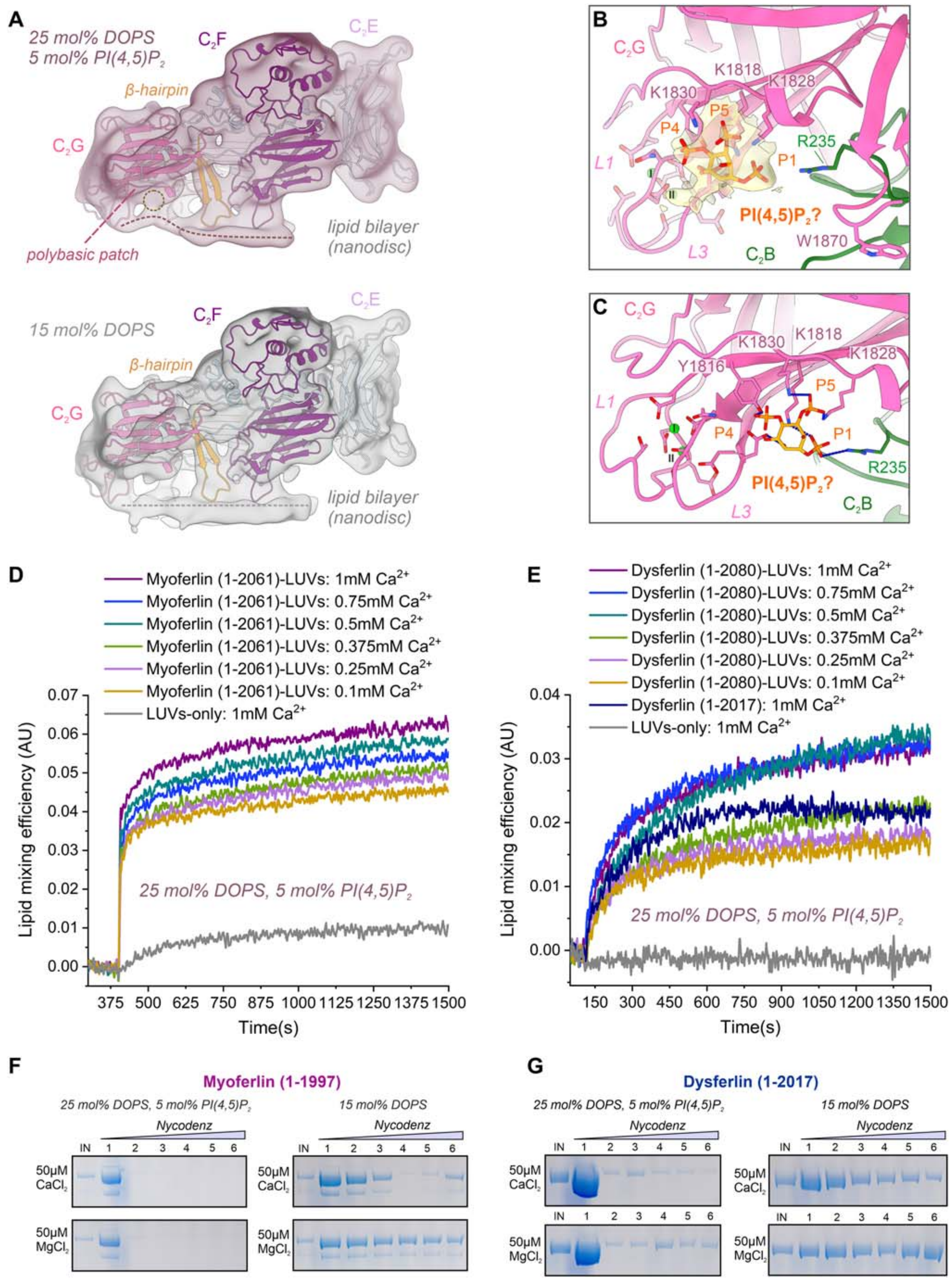
A
25 mol% DOPS
5 mol% PI(4,5)P2
C2F
C2E
β-hairpin
C2G
polybasic patch
lipid bilayer (nanodisc)
15 mol% DOPS
B
K1818
K1830
K1828
P5
P4
R235
P1
L1
PI(4,5)P2?
W1870
L3
C2B
C
Y1816
D
Myoferlin (1-2061)-LUVs: 1mM Ca2+
Myoferlin (1-2061)-LUVs: 0.75mM Ca2+
Myoferlin (1-2061)-LUVs: 0.5mM Ca2+
Myoferlin (1-2061)-LUVs: 0.375mM Ca2+
Myoferlin (1-2061)-LUVs: 0.25mM Ca2+
Myoferlin (1-2061)-LUVs: 0.1mM Ca2+
LUVs-only: 1mM Ca2+
Lipid mixing efficiency (AU)
Time(s)
25 mol% DOPS, 5 mol% PI(4,5)P2
E
Dysferlin (1-2080)-LUVs: 1mM Ca2+
Dysferlin (1-2080)-LUVs: 0.75mM Ca2+
Dysferlin (1-2080)-LUVs: 0.5mM Ca2+
Dysferlin (1-2080)-LUVs: 0.375mM Ca2+
Dysferlin (1-2080)-LUVs: 0.25mM Ca2+
Dysferlin (1-2080)-LUVs: 0.1mM Ca2+
Dysferlin (1-2017): 1mM Ca2+
LUVs-only: 1mM Ca2+
F
Myoferlin (1-1997)
Nycodenz
IN 1 2 3 4 5 6
50μM CaCl2
50μM MgCl2
G
Dysferlin (1-2017)

 © The Author(s)

Figure EV4. Full-length myoferlin and dysferlin appear to promote tight binding between vesicles.

(**A**) Comparison of the local nanodisc structures observed in the myoferlin (1–1997)-nanodisc complexes. The lipid nanodisc forms close contacts with the concave surface of C_2G in the presence (top, 25 mol% DOPS, 5 mol% PI(4,5)P_2, and 5 mol% cholesterol nanodisc, Appendix Fig. S4A–E), but not in the absence of PI(4,5)P_2 (bottom, 15 mol% DOPS and 5 mol% cholesterol nanodisc, Appendix Fig. S5C). The cryo-EM maps have been lowpass filtered to 10 Å, and the myoferlin model is fitted inside. (**B**) The cryo-EM density element proximal to C_2G could accommodate a PI(4,5)P_2 headgroup. C_2G and C_2B residues located at the interface are displayed as sticks. The putative PI(4,5)P_2 density lies in close proximity to three lysine residues (K1818, K1828, and K1830), projecting from the concave surface of C_2G, as well as to an arginine residue of C_2B (R235). The Ca^{2+}-binding L3 loop of C_2G is also in close proximity. (**C**) Polar contacts between several basic residues of C_2G and C_2B and a tentative phospholipid, possibly PI(4,5)P_2, originating from the nanodisc bilayer. The nanodisc contained 25 mol% DOPS and 5 mol% PI(4,5)P_2. (**D**) Lipid mixing assays between myoferlin proteoliposomes and PS/PI(4,5)P_2-bearing vesicles in the presence of varying Ca^{2+} concentrations. The fluorescent liposomes (Large unilamellar vesicles (LUVs), 25 mol% DOPS and 5 mol % PI(4,5)P_2) were dual-labelled with the Lissamine Rhodamine B and NBD (Nitrobenzoxadiazole). The NBD dequenching signal was used to monitor the extent of tight vesicle-vesicle docking (and, possibly, fusion) to non-fluorescent proteoliposomes. LUVs lacking full-length myoferlin (1–2061) were used as a control. Fluorescence traces were smoothed (using the Savitzky-Golay method) and normalized to the maximal dequenching signal to calculate the lipid mixing efficiency. The initial fluorescence increase is likely due to ferlin-induced aggregation of proteoliposomes. The lipid mixing assays were performed in triplicate, and representative fluorescence traces are shown. (**E**) Lipid mixing assays between dysferlin proteoliposomes and PS/PI(4,5)P_2-containing vesicles. The ability of full-length dysferlin (1–2080) to promote tight vesicle-vesicle docking was assessed as a function of Ca^{2+} concentration. The lipid mixing activity of soluble dysferlin (1–2017) was also tested (deep blue). Empty LUVs were used as a control. All assays were repeated at least three times, and representative fluorescence traces are shown. (**F**, **G**) Ca^{2+}-dependent liposome binding activity of soluble myoferlin (1–1997) and dysferlin (1–2017) assessed using a coflotation assay. The liposomes used in these assays had a similar lipid composition to those in (**D**, **E**). The Nycodenz step gradients (0%/30%/40%) were harvested from the top and analysed by SDS-PAGE. Both myoferlin (1–1997) and dysferlin (1–2017) showed increased binding to LUVs containing both DOPS and PI(4,5)P_2. Source data are available online for this figure.

© The Author(s)
